# Recent advances in g-C_3_N_4_ based sustainable sensors for toxic mercury (Hg^2+^) detection

**DOI:** 10.1039/d6ra01001f

**Published:** 2026-05-26

**Authors:** Suman Swami

**Affiliations:** a Department of Chemistry, University Institute of Sciences, Chandigarh University Mohali-140413 Punjab India sumanswami1994@gmail.com suman.e13442@cumail.in

## Abstract

Mercury (Hg^2+^) is an exceedingly toxic environmental pollutant, and its prolonged exposure poses a significant risk to human health and the ecosystem. The serious health risks associated with mercury exposure have been studied *via* regular monitoring of mercury in aquatic environments using different methods. Although the advantages of traditional methods are undeniable, they are often associated with some limitations. In response to these limitations, in recent years, nanomaterial-based sensing platforms have been developed as promising alternatives. Among the diverse nanomaterials, graphitic carbon nitride (g-C_3_N_4_) has captured substantial attention as an advanced sensing material owing to its distinctive band structure, excellent chemical and thermal stability, and versatile surface chemistry. The purpose of this review article is to provide the scientific community, researchers and scholars with a thorough overview of recent advances in g-C_3_N_4_-centered materials and their sensing mechanisms toward Hg^2+^ detection. It encompasses a diverse range, including g-C_3_N_4_ NPs, g-C_3_N_4_@metal nanoparticles, g-C_3_N_4_@metal oxides, g-C_3_N_4_@quantum dots, and g-C_3_N_4_@polymeric composites, elucidating structure–property relationships, sensing mechanisms, analytical performance, and real-sample applicability.

## Abstract 2

Mercury (Hg^2+^) is an exceedingly toxic environmental pollutant, and its prolonged exposure poses a significant risk to human health and the ecosystem.

## Graphical abstract

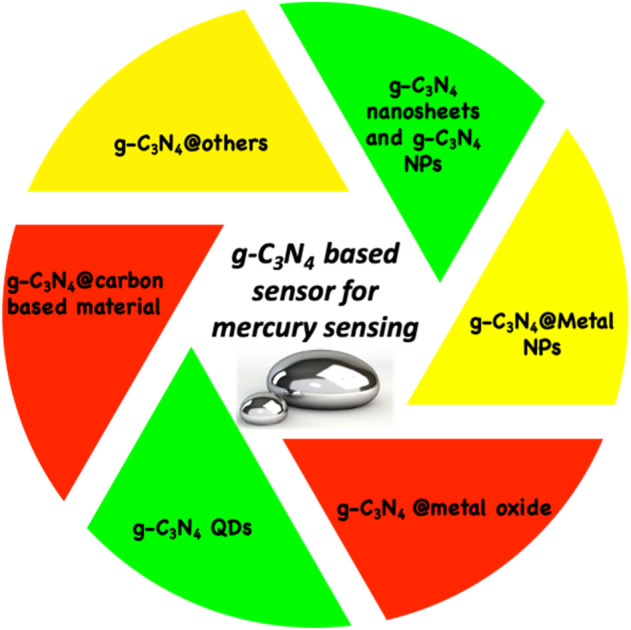

## 1. Introduction

Globally, the accessibility of safe and clean water is a vital necessity. Conversely, rapid industrialization and urbanization have resulted in severe contamination^1^ of natural water sources with various toxic metal ions.^2^ Especially, heavy metal ions (HMIs), such as lead (Pb), mercury (Hg), chromium (Cr), cadmium (Cd), and arsenic (As), are of particular concern due to their persistence, bioaccumulation and high toxicity profile.^3^ HMIs enter the ecosystem *via* effluents from the mining industry, industrial waste, paints, corroded metal pipelines, and other sources.^4,5^ As per a WHO report, ∼30% of the global population consumes water contaminated with toxic substances.^6^ When comparing HMIs, it is evident that mercury is highly toxic to human health due to its high water solubility,^7^ bioavailability and severe neurotoxicity, and it poses profound developmental risks during in utero and early childhood periods.^8–10^

Elemental mercury, a silvery liquid at room temperature, readily vaporizes under ambient conditions, contributing to its atmospheric mobility.^11^ Although the concentration of mercury in the environment is comparatively low, its high toxicity and long atmospheric residence time (0.8–1.7 years) make it a more serious global pollutant.^12^ Mercury contamination originated from both natural sources (volcanic activity and forest fires) and anthropogenic activities,^13^ including gold and silver extraction, dental amalgams, thermometers, electrical switches, battery manufacturing and chloro-alkali industries.^14–16^ Exposure of humans to mercury mainly occurs *via* drinking water and the food chain, especially through contaminated fish and seafood.^17,18^ Prolonged exposure to Hg^2+^ can be associated with many severe health issues, such as kidney and brain damage, neurological disorders, cardiovascular dysfunction, genetic mutation, teratogenesis, disruption in the cell inheritance, and increased risk of carcinogenesis.^19–22^ Moreover, inorganic mercury can be transformed into organic forms, which are considerably more toxic due to their high affinity to thiol (–SH) groups in proteins and enzymes,^23^ which further intensifies their biological impact. So, in view of the toxicological profile of mercury, the WHO established an acceptable limit of 30 nM^24,25^ for Hg^2+^ ions in drinking water, while the US-EPA has stipulated a more stringent limit of 10 nM (ref. 26) in drinking water, along with a maximum acceptable level of 0.3 ppm (0.3 mg kg^−1^) in fish and crab.^27^ Additionally, mercury has been included by WHO in their Global Oral Health Action Plan (2023–2030), which established a target that by 2030, 90% of countries will have implemented policies to reduce the use of dental amalgam, in line with the Minamata Convention on Mercury, or completely phase it out by 2030.^28^ Given this life-threatening risk, accurate, regular monitoring of mercury in water sources is essential. Numerous techniques have been devised for Hg(ii) ion detection, including spectrophotometry,^29^ atomic absorption spectrometry,^30^ X-ray fluorescence spectrometry,^31^ inductively coupled plasma mass spectrometry,^32^ Rayleigh light-scattering,^33^ potentiometry,^34^ gas chromatography-mass spectrometry,^35^ and neutron activation analysis.^36^ Despite the high accuracy and undeniable reliability of these methods, there remains a sustained demand for simple and sensitive technologies that can be used for off- and online monitoring of Hg(ii) content in real samples for practical analytical applications. In recent years, many sensing materials, including organic chemosensors^37,38^ and nanomaterials,^39^ have been employed for sensing of toxic mercury ions. Compared to chemosensors, which often require complex synthesis and organic solvents, carbon-based nanomaterials provide a paradigm shift in sensor development owing to their easy synthesis, high surface area and tunable optical properties. Graphitic carbon nitride (g-C_3_N_4_) has appeared as a promising material owing to its unique electronic band structure, high chemical and thermal stability, versatile surface chemistry, and catalytic properties. Like graphite, g-C_3_N_4_ has a layered structure involving weak van der Waals interactions between adjacent C–N layers.^40^ It has been successfully utilized in solar energy transfer, water splitting, pollutant degradation, photocatalysis, sensors, lithium batteries and fuel cells, owing to its excellent biocompatibility, outstanding photostability and low toxicity.^41^ The optical properties, explicitly blue photoluminescence (430–550 nm) with a maximum at ∼470 nm, superior surface area, enhanced fluorescence quantum yield, excellent aqueous dispersibility and activated interactive binding sites, mean that g-C_3_N_4_-based sensors hold significant potential for easy and sustainable detection of mercury in various environmental samples. A variety of review articles have examined the sensing of HMIs^42–45^ and mercury sensing;^46–48^ for instance, in 2023, Qiuping Li and You Zhou reviewed fluorescent materials that have been explored for Hg^2+^ detection;^49^ in 2024, Mohamed J. Saadh *et al.* reviewed quantum dot based sensors for Hg^2+^ sensing;^50^ in 2024, Frank Tukur *et al.* reviewed SERS and IIPs based Hg^2+^ detection.^51^ Recently, in 2025, Ansh Jaswal *et al.* reviewed coumarin-derived fluorescent chemosensors for Hg^2+^ detection,^21^ and Imran Muhammad *et al.* reviewed fluorophore-based fluorescent sensors for Hg^2+^ detection.^52^ However, no review has focused on g-C_3_N_4_-based sensors for mercury sensing as a single platform. Despite extensive literature on mercury sensing, the prospects of g-C_3_N_4_ sensors for mercury detection remain significantly unexplored. Therefore, the present review article aims to provide the scientific community, researchers and scholars with an inclusive view of recent advances in g-C_3_N_4_-derived materials and their sensing mechanisms toward Hg^2+^ detection.

## 2. Synthesis and properties of g-C_3_N_4_

**Fig. 1:**
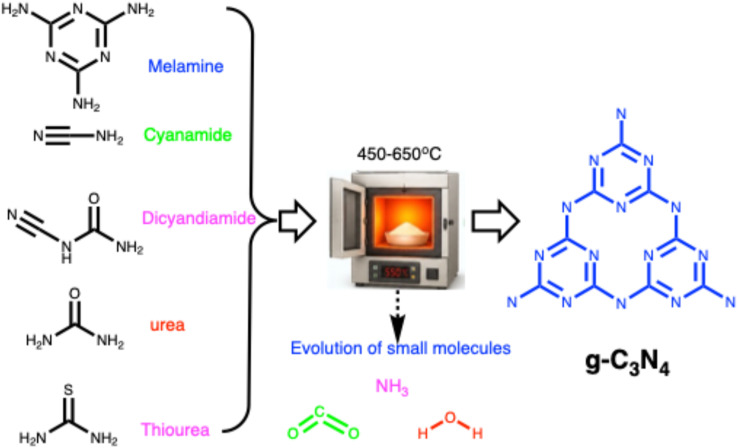
g-C_3_N_4_ synthetic routes: thermal condensation of nitrogen-rich precursors (melamine, cyanamide, dicyandiamide, and urea, thiourea) at specific temperature ranges (450–650 °C).

Carbon nitrides (C_3_N_4_), a unique class of metal-free semiconductors with structural similarity to graphite, have attracted remarkable attention over the past decade.^53^ These polymeric materials consist primarily of carbon and nitrogen atoms.^54^ The development of carbon nitride dates back to 1834, when Liebig reported a linear polymer named “melon”, composed of linked tri-*s*-triazine units connected *via* secondary nitrogen atoms.^55^ Despite its discovery nearly two centuries ago, the potential of this polymer-like material remained underexplored for many years due to its insolubility, chemical inertness, high thermal stability, and initially unclear structure. Later, in 1996, Teter and Hemley proposed five distinct crystalline phases of C_3_N_4_ α-, β-, cubic-, pseudocubic-, and graphitic-C_3_N_4_ (g-C_3_N_4_).^56^ Subsequent experimental and theoretical studies confirmed that the tri-*s*-triazine-based g-C_3_N_4_ is the most thermodynamically stable phase, being approximately 30 kJ mol^−1^ more stable than the *s*-triazine-based structure.^57^ The tri-*s*-triazine or heptazine unit is now extensively recognized as the ultimate building block of g-C_3_N_4_.^58^ Characteristically, g-C_3_N_4_ is synthesized *via* thermal condensation of various nitrogen-rich precursors (urea, thiourea, melamine, cyanamide, and dicyandiamide) at specific temperature ranges (450–650 °C) under air or inert conditions^59^ (Fig. 1). The degree of polymerization of the tri-*s*-triazine framework in g-C_3_N_4_ changes considerably, depending on the thermal treatment temperature,^60^ which directly impacts the density and nature of the active nitrogen sites in the material. At lower temperatures (up to ∼350 °C), the products are primarily melamine-based. Around 390 °C, melamine undergoes structural rearrangement to form tri-*s*-triazine units. Further condensation of these units leads to the formation of g-C_3_N_4_ polymeric networks near ∼520 °C, while the material begins to lose stability slightly above 600 °C.^61^ When temperatures reach 700 °C, complete thermal decomposition occurs, resulting in residue-free disappearance through the evolution of –N and –CN fragments. At lower synthesis temperatures (∼450 °C), polymerization remains incomplete, producing partially condensed structures with a high content of terminal amino groups (–NH_2_, –NH–), increased structural defects, and low crystallinity. Although such materials possess a high density of defect-associated nitrogen sites, these sites are less electronically delocalized and exhibit weaker coordination interactions with Hg^2+^ ions. The use of intermediate temperatures (500–550 °C) delivers well-developed tri-*s*-triazine frameworks with extended π-conjugated networks, abundant sp^2^-hybridized nitrogen (C=N–C) sites and balanced defect density. These nitrogen centers act as strong electron donors, significantly enhancing coordination affinity toward Hg^2+^ ions and thereby improving sensing performance. At higher temperatures (600–650 °C), due to over-condensation and partial thermal decomposition, nitrogen density decreases *via* volatilization (*e.g.*, NH_3_ release), which results in fewer surface functional groups and a reduction in defect density, ultimately decreasing the number of accessible active sites for Hg^2+^ binding, despite improved crystallinity.^62^ The choice of precursor and calcination conditions strongly influences the physicochemical properties of the resultant material, including porosity, band gap, photoluminescence, and surface charge. Theoretical computations imply that, among the various possible crystalline forms, g-C_3_N_4_ is the most stable allotrope owing to its graphite-like layered structure. Graphitic carbon nitride exhibits an optical band gap of ∼2.7 eV, which can be tuned up to 5 eV depending on the degree of condensation.^63,64^ It also displays strong photoluminescence, with emission maxima between 366 and 472 nm, related to electronic coupling between adjacent sheets. Due to its tunable optical and electronic characteristics, g-C_3_N_4_ has found wide application in photocatalysis, sensing (HMIs, antibiotics, and phenolic compounds) and solar energy conversion. However, bulk g-C_3_N_4_ generally exhibits a low surface area and a limited number of active sites, restricting its catalytic and sensing performance. Moreover, the abundant nitrogen-containing functional groups (*e.g.*, primary and secondary aromatic amines) in the g-C_3_N_4_ framework facilitate surface modification and adsorption processes. Therefore, researchers have explored a category of nanostructured forms such as nanotubes, nanofibers, mesoporous frameworks, and ultrathin nanosheet CDs, QDs and NPs derived from g-C_3_N_4_ to offer superior surface area, enhanced fluorescence quantum yield, and excellent aqueous dispersibility, making them particularly attractive for advanced applications.

## 3. g-C_3_N_4_-centered composites for mercury sensing

**Fig. 2:**
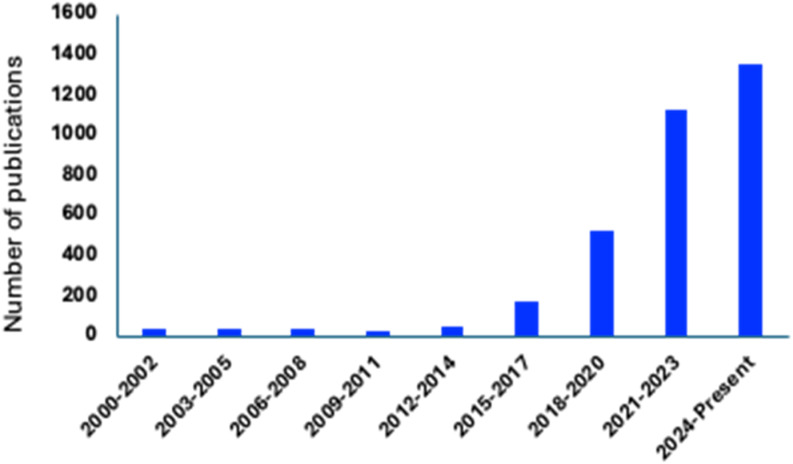
Progress in publications: g-C_3_N_4_-centered sensors for environmental analyte sensing (data grouped from Google Scholar, Research Gate, Science Direct, and PubMed Central).

**Fig. 3:**
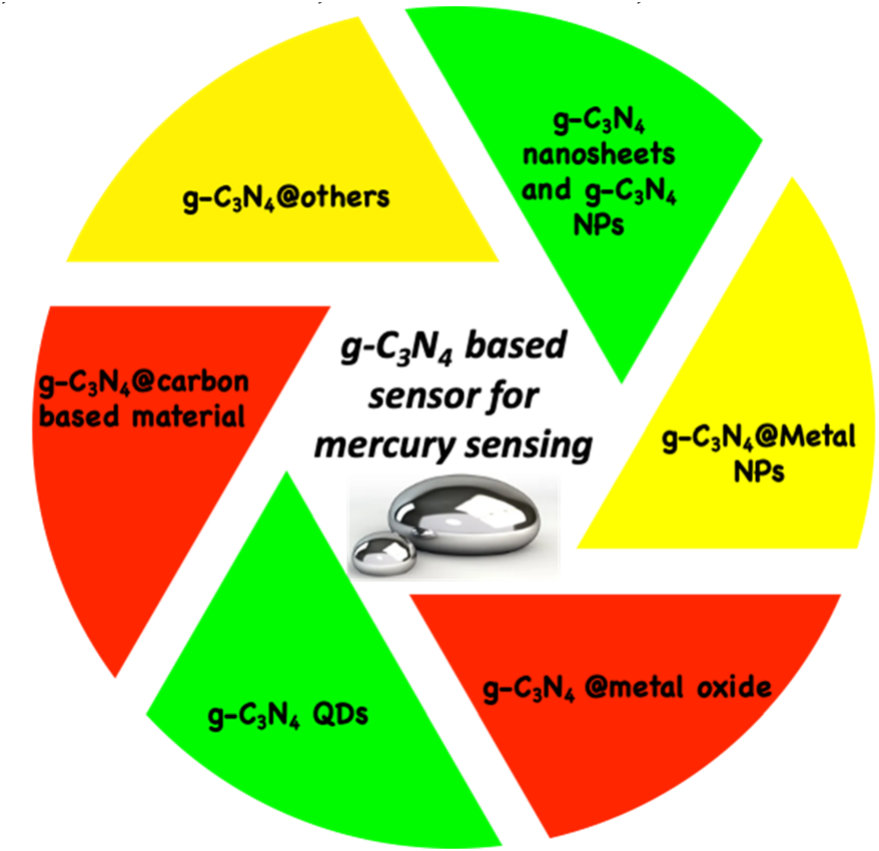
g-C_3_N_4_-centered composites for mercury sensing.

Over the years, g-C_3_N_4_-based nanocomposites and nanomaterials have emerged as robust materials that have been used for the development of green, efficient and sustainable sensing probes for environmental analytes (heavy metal ions, antibiotics, and endocrine disruptors, *etc.*) (Fig. 2). The high affinity for mercury ions, tunable electronic and optical properties, high surface area, porous structure, excellent stability, and biocompatibility, along with its straightforward and low-cost synthesis, render g-C_3_N_4_ a versatile and effective material for the development of sensing platforms for mercury ions. Owing to its possible integration with different classes of materials (NPs, metal oxides, CDs, QDs, polymeric material, *etc.*), a diverse range of g-C_3_N_4_-centered composites have been used for mercury ion sensing (Fig. 3).

### 3.1 g-C_3_N_4_ nanosheets and g-C_3_N_4_ NP sensors for mercury sensing

**Fig. 4:**
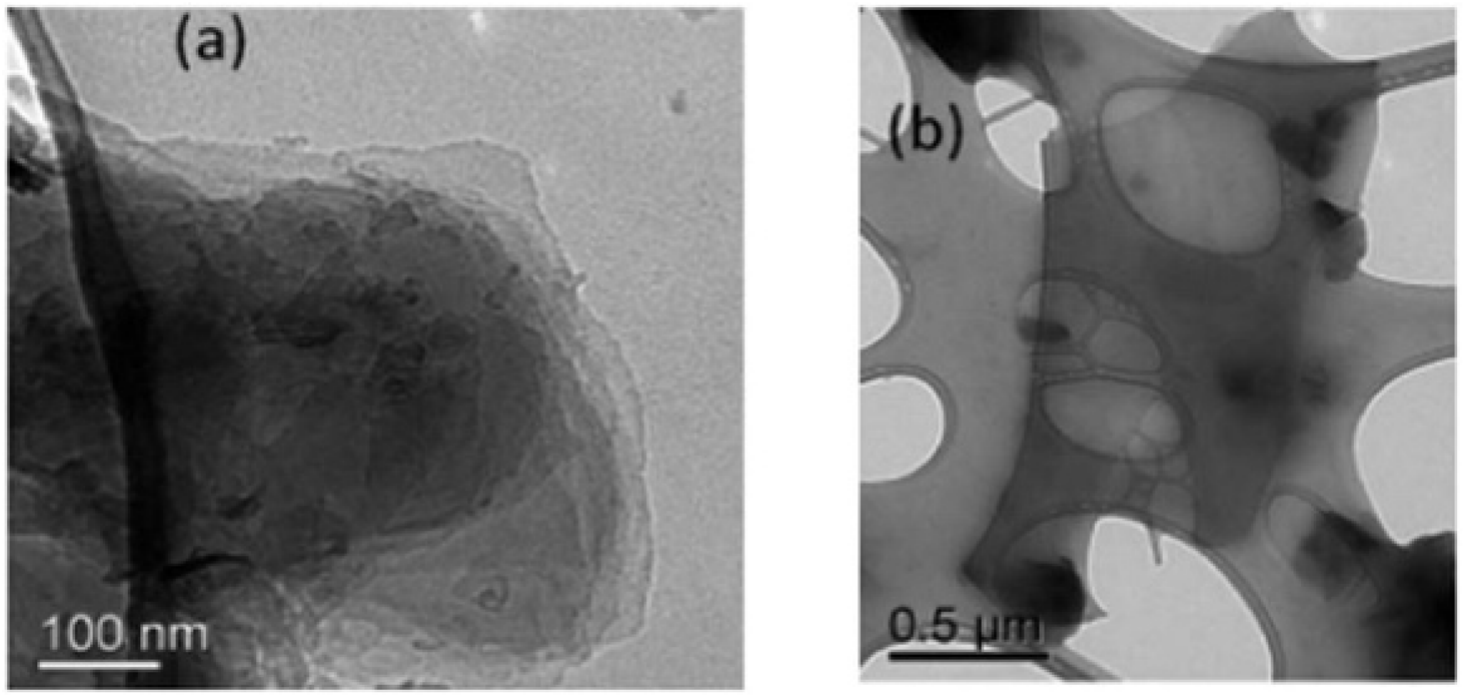
TEM images of g-C_3_N_4_ sheets (a) with scale bars of 100 nm and (b) 0.5 µm. This figure has been adapted from ref. 65 with permission from RSC, copyright 2013.

Graphitic carbon nitride (g-C_3_N_4_) nanosheets and nanoparticles have appeared as promising platforms for mercury sensing due to their high surface area, excellent chemical stability, and tunable electronic properties. For instance, two-dimensional g-C_3_N_4_ nanosheets, prepared *via* microwave-assisted (MW) formamide pyrolysis, have been reported to provide an effective platform for mercury sensing. TEM analysis confirmed layer-by-layer stacked morphology (Fig. 4), while the XRD pattern revealed a sharp and intense (002) reflection at 27.26 (*d* = 3.27 Å), consistent with graphitic carbon nitride. Such structural features provide a large surface area and abundant active sites for favorable electrochemical applications. When g-C_3_N_4_ is immobilized on a glassy carbon electrode, the g-C_3_N_4_ enables sensitive electrochemical detection of mercury ions in an aqueous medium. Notably, the g-C_3_N_4_-modified system allowed concurrent detection of Pb^2+^, Cu^2+^ and Hg^2+^, indicating multi-metal sensing capability. The stripping peak current is strongly dependent on the potential and accumulation time, underscoring the preconcentration parameters. Interestingly, the sensor exhibits a remarkable response in the acidic pH range (pH 1–6) while it is reduced in alkaline media. This limitation suggested that environmental samples with higher pH required pretreatment before analysis. The optimized protocol involved accumulation and reduction of Hg^2+^ to Hg^0^ at −1.0 V for 15 minutes in 0.02 M HCl, followed by CV scanning between 0–0.6 V. Under these optimized conditions, a limit of detection (LOD) of 9.1 × 10^11^ M was achieved, which was three orders below the WHO drinking water limit (10^8^ M). Such an ultra-trace LOD (9.1 × 10^11^ M) enables the potential application of g-C_3_N_4_ nanosheets in environmental monitoring. However, the relatively long accumulation time (15 min) and narrow working pH range (pH 1–6) may limit practical field deployment for wider sample analysis.^65^

Furthermore, ultrathin C_3_N_4_ nanosheets have been developed by exfoliating bulk g-C_3_N_4_, which was itself synthesized *via* a thermal polycondensation route. Structural characterization by TEM, XRD, FTIR and AFM established a graphite-like layer morphology with a nanosheet thickness of ∼8 nm. TEM images revealed that bulk g-C_3_N_4_ appears as irregular thick aggregates, whereas the exfoliated material exhibits nearly transparent sheet-like features, confirming successful ultrathin nanosheet formation. Electrochemical sensing studies demonstrated that utg-C_3_N_4_-modified glassy carbon (GC) electrodes significantly outperformed their bulk counterparts. Under the optimized conditions, anodic stripping voltammetry (ASV) shows a linear response to Hg^2+^ over the range of 0.1–15 µg L^−1^, achieving a remarkable LOD of 0.023 µg L^−1^ and 6.8 µA (µg L^−1^)^−1^ cm^−2^ sensitivity. The enhanced response is attributed to the strong interaction between utg-C_3_N_4_ and Hg^2+^ through its –NH and –NH_2_ groups, which facilitate efficient binding and electron transfer. Importantly, the sensor was validated in real samples, including lake water, tap water, and river water, with satisfactory recovery values. The low LOD and its applicability in real samples highlight the potential application of utg-C_3_N_4_ for environmental monitoring. However, despite the excellent analytical performance of utg-C_3_N_4_, the need for laborious exfoliation steps and possible reproducibility issues during nanosheet preparation may pose challenges for large-scale production.^66^

**Fig. 5:**
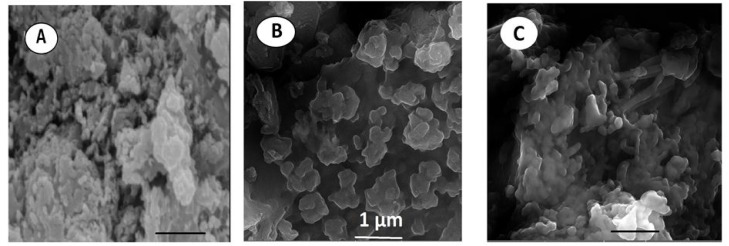
FESEM images (scale bar 1 µm) of (A) bulk, (B) nanosheets, and (C) carboxylated g-C_3_N_4_. This figure has been adapted from ref. 63 with permission from Elsevier, copyright 2016.

Carboxyl functionalized g-C_3_N_4_ nanoparticles (carboxy–g-C_3_N_4_) have been synthesized *via* one-pot thermal polymerization of dicyandiamide under a nitrogen atmosphere, followed by surface modification. Structural characterization by XRD, FESEM, AFM, and FTIR confirmed the successful formation of nanosheets with a lateral size of 35–50 nm and a thickness of ∼6 nm (Fig. 5). The sensing ability of carboxylated g-C_3_N_4_ was evaluated using photoluminescence (PL) intensity changes. Carboxyl–g-C_3_N_4_ exhibited pronounced fluorescence quenching in the presence of Fe^3+^ and Hg^2+^, whereas other ions induced negligible effects, confirming its sensitivity to Fe^3+^ and Hg^2+^. The enhanced response toward Hg^2+^ and Fe^3+^ was attributed to stronger binding affinity and faster chelation kinetics with N and O groups present in the carboxylate framework. Structural considerations further support this sensitivity: the pore size of carbon nitride (0.7 nm) and the hydrate diameter of Hg^2+^ (0.5 nm) and Fe^3+^ (0.9 nm) facilitate ready diffusion of the mercury ions into the pores and capture by the donor sites (nitrogen and oxygen). Mechanistically, the interaction between Hg^2+^ and carboxy–g-C_3_N_4_ involves covalent bonding (Hg–N) alongside the electrostatic interaction, leading to fluorescence static quenching. In contrast, Fe^3+^ quenching was weaker, likely due to steric limitations in the pore mechanism; this suggests that carboxylate g-C_3_N_4_ is highly selective for Hg^2+^, outperforming many conventional nanomaterials. However, although this sensor has convincing dual Hg^2+^/Fe^3+^ detection applications, certain limitations remain challenging; specifically, the strong PL quenching response to Fe^3+^ may complicate selective mercury detection in mixed ion systems unless additional separation or masking strategies are applied.^63^ For instance, J. Wang *et al.* explored the use of sodium diethyldithio carbamate (DDTC) as a masking agent with 1,2-dithioglycol functionalized-CNQDs for fluorescence sensing of Hg^2+^, given that, in their work, Cu^2+^ and Ag^+^ also exhibited quenching to some extent. Studies in the presence of the masking agent DTG-CNQDs indicate that fluorescence quenching was mainly caused by Hg^2+^, and other ions had almost no influence on the fluorescence.^67^ Furthermore, diethanolamine (DEA) and thioglycolic acid TGA were used as efficient masking reagents on g-C_3_N_4_-ssDNA with the purpose of eliminating interference by Cu^2+^ and Fe^3+^. In addition, the influence of Ag^+^ could be easily removed by adding Cl^−^. When numerous types of interfering ions were present, the fluorescence intensity revealed that the sensor was relatively selective towards Hg^2+^ metal ions.^68^ Similarly, to evaluate the selectivity of BCN toward the Hg^2+^ and Fe^3+^ ions, the emission spectra of the BCN suspension were monitored in the presence of each ion (Hg^2+^ and Fe^3+^) and larger amounts of competing cations. Both Hg^2+^ and Fe^3+^ ions created a significant quenching in the fluorescent emission of BCN in the presence of other competing metal ions. However, both Fe^3+^ and Hg^2+^ ions had similar quenching effects, so to distinguish Hg^2+^ and avoid interference from Fe^3+^, a masking agent, *i.e.* sodium hexametaphosphate (SHMP), was added. In the presence of SHMP, the quenching by Fe^3+^ ions is suppressed, whereas quenching by Hg^2+^ ions remain, providing a simple way to distinguish between the Hg^2+^ and Fe^3+^ ions; BCN can be regarded as a selective probe for the detection of Fe^3+^ and Hg^2+^ ions in aqueous solutions.^69^

**Scheme 1:**
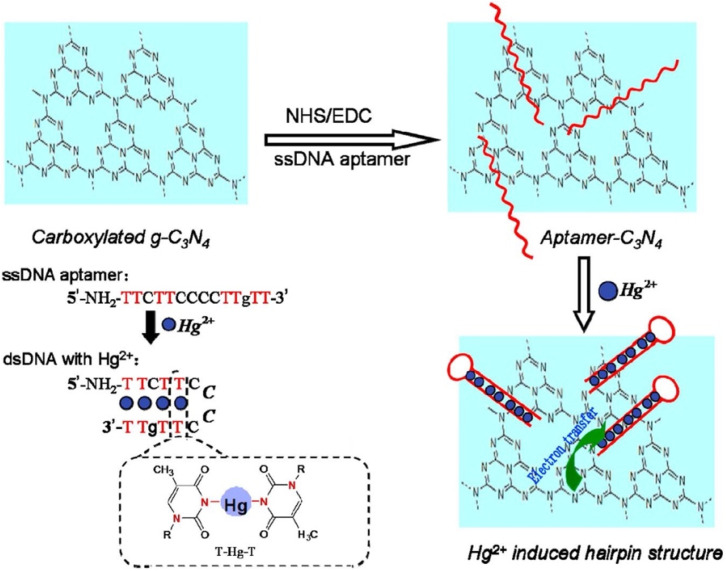
Schematic of Hg^2+^ detection based on the fluorescence quenching of g-C_3_N_4_. This figure has been adapted from ref. 68 with permission from Elsevier, copyright 2016.

Duan *et al.* reported a facile g-C_3_N_4_ nanosheet-based fluorescent platform utilizing an “on–off–on” approach for dual detection of 6-thioguanine (6-TG) and Hg^2+^. In the absence of Hg^2+^, the addition of 6-TG to the solution of g-C_3_N_4_ nanosheets quenched the intrinsic fluorescence of g-C_3_N_4_ nanosheets *via* hydrogen bonding and pi–pi stacking interaction. Upon subsequent addition of Hg^2+^, the quenched fluorescence of g-C_3_N_4_ nanosheets was restored due to preferential binding of Hg^2+^ with 6-TG, which disrupted molecular interactions between 6-TG and the g-C_3_N_4_ surface. This dual-mode sensing system achieved quantitative detection of 6-TG with a LOD of 65 nM. Meantime, the same platform functioned as a “turn on” sensor for Hg^2+^ with a LOD of 37 nM. Practical applicability was validated in spiked tap water and spring water samples with good recoveries ranging from 95.2% to 98.6%, and the relative standard deviations were 2.3% and 4.2%, confirming the reliability of the method in complex mixtures. The advantage of this strategy is its versatility, wherein the same g-C_3_N_4_ nanosheet system enables both pharmaceutical monitoring of 6-TG, an anticancer drug, and heavy-metal sensing (Hg^2+^). Moreover, the relatively low LOD makes it competitive for environmental analysis. However, the system accomplishes sensing *via* specific affinity between Hg^2+^ and 6-TG; thus, in real environmental samples containing thiol-rich organic compounds, interference could reduce the selectivity. Additionally, while fluorescence restoration provides a convenient readout, the inherent dependence on a dual-analyte system may limit its practical deployment compared to simpler single-analyte probes.^70^ Similarly, a DNA-aptamer-based fluorescent biosensor has been established using g-C_3_N_4_ nanosheets as a platform for selective sensing of Hg^2+^. In the strategy, the g-C_3_N_4_ sheets were functionalized with a single-stranded DNA (ssDNA) aptamer that exhibits strong fluorescence emission at 440 nm upon excitation at 380 nm in the absence of Hg^2+^. The addition of Hg^2+^ induced the formation of a hairpin-shaped double-stranded DNA (dsDNA) structure. This conformational change brought Hg^2+^ ions into proximity with the surface of the g-C_3_N_4_ sheet, as depicted in Scheme 1, resulting in efficient fluorescence quenching. The biosensor demonstrated a linear response over an inclusive range (0.5–100 µM) with a remarkably low LOD of 0.17 nM, which is almost two orders lower than the U.S. EPA guideline for Hg^2+^ in drinking water (10 nM). The practicality of the sensor was validated through recovery studies in spiked tap water samples, yielding recoveries of 98.3% to 110.8%, and the RSD was in the range of 2.6–4.7%, confirming its high reliability in complex matrices. The key strength of this design lies in the synergy of the aptamer selectivity with the high surface area and fluorescence efficiency of g-C_3_N_4_, enabling ultrasensitive detection well below regulatory limits. Moreover, the biosensor's performance in real water samples underscores its translational potential for environmental monitoring. However, the approach requires careful synthesis and stabilization of aptamer-g-C_3_N_4_ conjugates, which may limit scalability. Additionally, in natural waters containing high levels of competing metal ions or nucleic-acid-binding contaminants, selectivity could be challenged despite the high affinity of the T-Hg^2+^-T motif.^68^

**Scheme 2:**
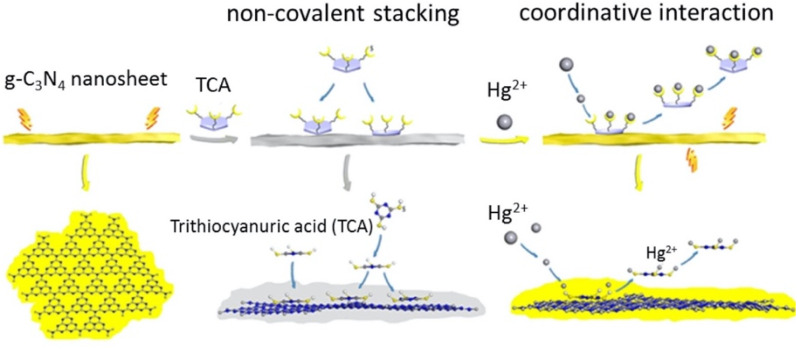
Schematic illustrating how the g-C_3_N_4_ nanosheets serve as fluorescent sensors for the detection of TCA and Hg^2+^. This figure has been adapted from ref. 71 with permission from Elsevier, copyright 2019.

**Fig. 6:**
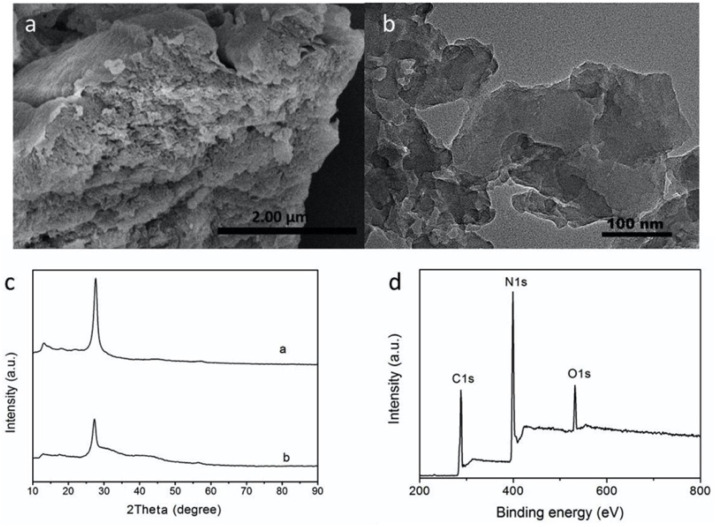
(a) SEM and (b) TEM images of g-C_3_N_4_ nanosheets. (c) XRD patterns of bulk g-C_3_N_4_ and g-C_3_N_4_ nanosheets, and (d) the survey spectrum of g-C_3_N_4_ nanosheets. This figure has been adapted from ref. 71 with permission from Elsevier, copyright 2019.

Similarly, Li *et al.* reported a multifunctional fluorescent nanosensor based on g-C_3_N_4_ nanosheets and trithiocyanuric acid (TCA), exploiting competitive interactions between noncovalent stacking (g-C_3_N_4_–TCA) and coordination (TCA–Hg^2+^). The nanosheets were successfully exfoliated from bulk g-C_3_N_4_, as established by XRD, SEM and TEM analysis, and showed a sheet-like monolayer structure. The XRD patterns further revealed the structural features of (100) and (002) planes for the g-C_3_N_4_ nanosheets, endorsing the effective exfoliation of bulk g-C_3_N_4_ (Fig. 6). In the sensing mechanism, the addition of TCA to a g-C_3_N_4_ nanosheet solution induced fluorescence quenching *via* H-bonding and pi–pi interactions. Upon introduction of Hg^2+^ ions, the fluorescence of the TCA–g-C_3_N_4_ nanosheet hybrid was effectively restored. This behaviour is attributed to the preferential coordination of Hg^2+^ with the sulfur atoms of TCA, which disrupts the pi–pi stacking interaction between TCA and g-C_3_N_4_, as illustrated in Scheme 2. This competitive binding enabled dual detection of TCA and Hg^2+^, with a LOD of 6.2 × 10^−7^ M for Hg^2+^. The study thus demonstrates the versatility of g-C_3_N_4_ for constructing multifunctional competitive interaction sensors that can simultaneously target organic pollutants and heavy metals. The advantage of this design lies in its ability to convert binding competition into a measurable fluorescence change, providing high selectivity in mixed systems. However, the sensing mechanism depends on indirect displacement (TCA–Hg^2+^ coordination), which could be influenced by competing sulfur- or nitrogen-containing species in natural waters, which reduced the specificity of the method under complex environmental conditions.^71^

**Scheme 3:**
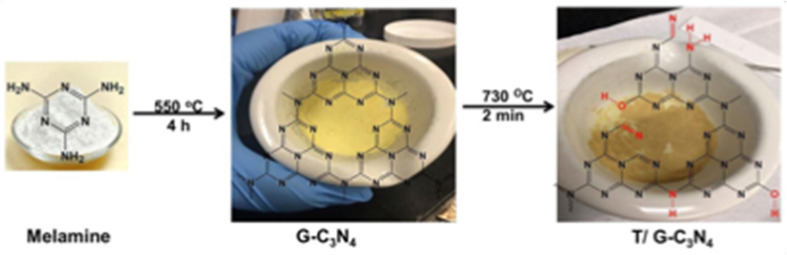
Synthesis of the T/G-C_3_N_4_ nanosheets.

A functionalized graphitic carbon nitride nanosheet (T/G-C_3_N_4_) fluorescence probe was fabricated *via* melamine pyrolysis followed by rapid KOH treatment. Spectroscopic and structural analysis confirmed the successful modification. Density functional theory (DFT) calculations further supported the stability of T/G-C_3_N_4_ and its strong interaction with Hg(ii). The sensors exhibited a quenching response over a linear range of 0 to 1.25 × 10^3^ nM Hg(ii) with a LOD of 27 nM. The sensing mechanism was attributed to the photoinduced electron transfer from the valence band to the conduction band of T/G-C_3_N_4_; consequently, after the interactions between T/G-C_3_N_4_ and Hg^2+^, electrons are transferred to the LUMO of the Hg^2+^ ions. This process resulted in fluorescence quenching due to the recombination of photoinduced electron–hole pairs. This system offers advantages, including simple preparation, a wide linear range, and theoretical validation of the sensing mechanism, which enhances credibility. But the real sample testing aspect was not extensively discussed, particularly with respect to selectivity in complex environmental matrices (Scheme 3).^72^

**Fig. 7:**
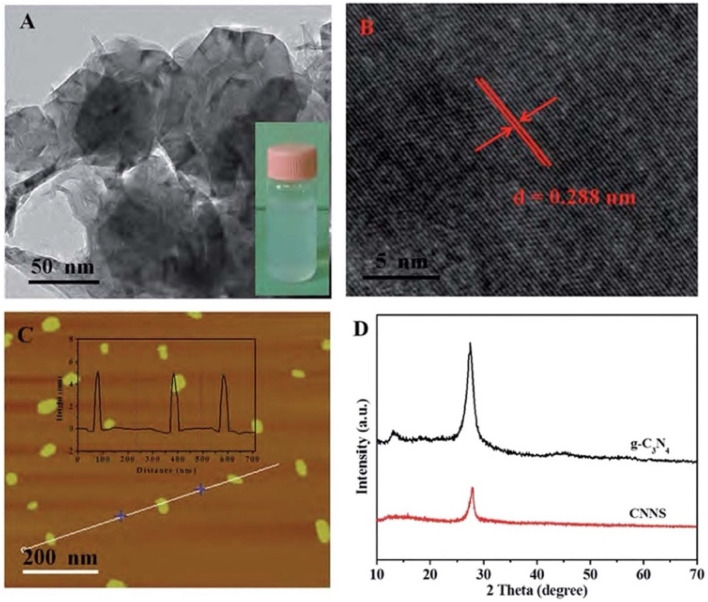
(A) TEM, (B) HR-TEM, and (C) AFM images of CNNS, and (D) XRD spectra of g-C_3_N_4_ and CNNS. This figure has been adapted from ref. 41 with permission from RSC, copyright 2015.

Comparably, H. Zhang *et al.* developed graphitic carbon nitride nanosheets (CNNS) *via* strong acid-assisted ultrasonic exfoliation of bulk g-C_3_N_4_. The structural and morphological features of CNNS were confirmed using TEM, AFM, XRD, XPS, FT-IR and fluorescence analyses. TEM images clearly revealed ultra-thin nanosheets with a lattice spacing of about 0.288 nm (Fig. 7B), while AFM indicated a thickness of approximately 5 nm, confirming the formation of a few layers of C–N sheets (Fig. 7C). In a fluorescence sensing study, the CNNS exhibited a concentration-dependent fluorescence quenching in response to Hg^2+^, indicating that the system was significantly responsive to Hg^2+^ concentration. Importantly, the CNNS enabled satisfactory sensitivity and selectivity for Hg^2+^ and l-Cys *via* the dual “off–on” mode. Hg^2+^ quenching occurred due to the covalent interaction between the empty orbital of Hg^2+^ and the p-electrons of N (turn-off) CNNS. Upon the introduction of l-Cys, fluorescence was restored, as the –SH group of l-Cys can interact with Hg^2+^ and drag it from the surface of the CNNS (turn-on). The method was validated for the determination of Hg^2+^ and l-Cys in real water samples with a recovery range of 97–102% and 97.3–103%, in tap and well water, respectively. The result signified that the present sensing platform could be efficiently applied for the determination of Hg^2+^ and l-Cys in real environmental samples. This sensing material is noteworthy for demonstrating a simple yet effective nanosheet-based “off–on” sensing platform capable of detecting both toxic metal ions and biologically relevant thiols. However, the dependence on strong acid treatment for exfoliation raises concerns regarding scalability and environmental friendliness.^41^

**Scheme 4:**
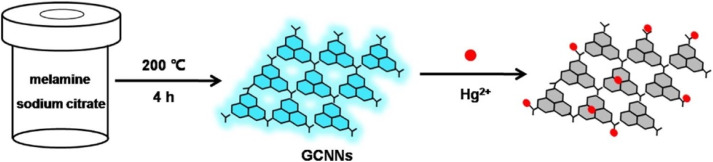
Schematic of Hg^2+^ sensing *via* fluorescence response. This figure has been adapted from ref. 73 with permission from Elsevier, copyright 2016.

**Fig. 8:**
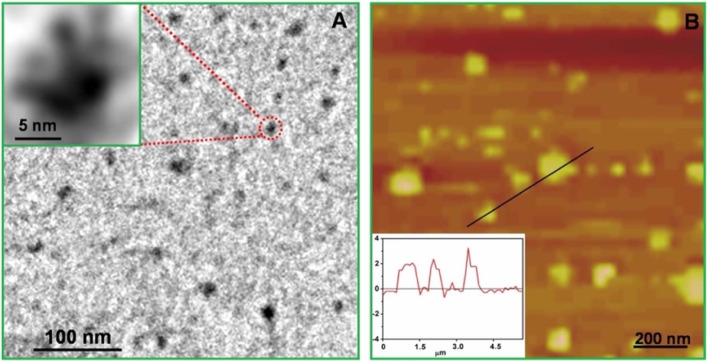
(A) HR-TEM (inset; enlarge image part) and (B) AFM images of GCNNs (inset; the height profile). This figure has been adapted from ref. 73 with permission from Elsevier, copyright 2016.

Q. Zhuang, L. Sun, and Y. Ni reported the development of highly fluorescent GCNNs for Hg^2+^ sensing, as depicted in Scheme 4. Structural analyses by TEM and AFM confirmed well-dispersed spherical particles (∼12.6 nm) with a few-layer thickness (2–3 nm), while the XRD pattern displayed a broad (002) peak at 27.8°, indicating poor crystallinity due to weak pi–pi stacking. Moreover, FTIR analysis suggested abundant surface functionalities (–COOH, –OH, –NH_2_), which are advantageous for metal ion coordination and fluorescence response. Importantly, fluorescence quenching by Hg^2+^ was highly selective compared to other cations, achieving an impressive LOD of 0.3 nM over a broad linear range (0.001–1.0 µM). All these results highlight the promise of GCNNs as sensitive probes for Hg^2+^ in real samples, such as water and milk; however, the study did not address potential interference from complex environmental metrics, long-term stability, or regeneration of the sensor. Although this work demonstrates the utility of surface functionalized GCNNs, further studies are required to establish robustness under practical field conditions (Fig. 8).^73^

**Fig. 9:**
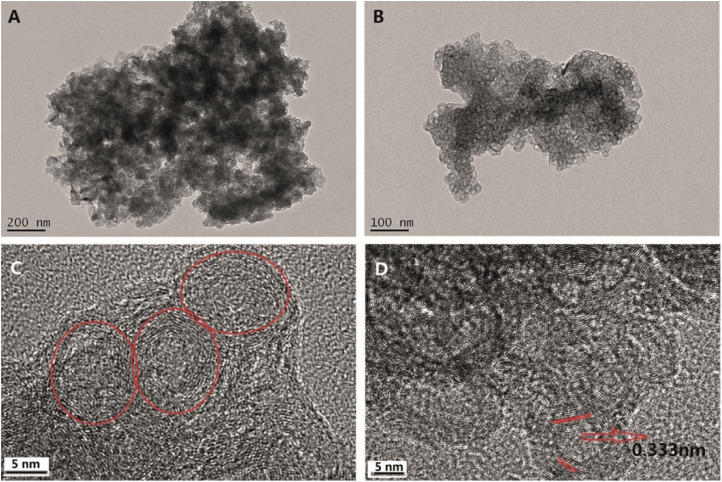
(A and B) TEM and (C and D) HRTEM images of as-obtained CNNPs. This figure has been adapted from ref. 74 with permission from Elsevier, copyright 2015.

A one-pot solvothermal strategy was explored by Ma *et al.* for the synthesis of fluorescent carbon nitride nanoparticles (CNNPs) using oleic acid as a green solvent. The resulting CNNPs displayed hollow cross-linked morphology, as confirmed by TEM (Fig. 9), and were extensively characterized using XRD, FTIR, XPS and solid-state NMR. The FTIR spectrum revealed characteristic C=N stretching bands at 1370 and 1400 cm^−1^, and a triazine breathing mode band at 764 cm^−1^, confirming the successful polymerization of nitrogen-rich structures. The CNNPs exhibited strong and selective fluorescence quenching towards Hg^2+^ ions with a LOD of 0.094 µM, which is lower than or comparable to many existing fluorescent probes. Interestingly, a dual linear response was observed across two distinct linear concentration ranges of 0.1–8 and 8–32 µM, signifying the presence of two different interaction sites on the surface of the nanoparticles for interaction of Hg^2+^ ions. At lower concentrations, Hg^2+^ ions favorably bind with high-affinity binding sites, resulting in rapid fluorescence quenching, whereas at higher concentrations, an additional weaker binding or aggregation process contributed. The proposed sensing mechanism involves Hg^2+^–nitrogen coordination, and its ability to form a stable non-fluorescent complex with the CNNPs and possible aggregate-induced quenching due to simultaneous binding of Hg^2+^ to multiple N and O sites. This complexation process alters the CNNP electronic structure, leading to enhanced non-radiative recombination. Further real sample analysis indicates that the prepared CNNPs exhibit satisfactory potential detection of Hg^2+^ ions in real samples. However, long-term photostability and performance in more complex matrices remain unaddressed, which limits immediate real-world deployment.^74^ Quenching mechanisms (static and dynamic) may co-exist in fluorescence-based sensing systems.^75^ Primarily static quenching arises due to a non-fluorescent ground-state complex formation between the fluorophore and the quencher, whereas dynamic quenching arises due to collisional interactions between the excited-state fluorophore and the quencher. The distinction between these mechanisms can be effectively established based on their dependence on temperature, viscosity, and, most reliably, through fluorescence lifetime measurements. For example, in the case of Ag–S–g-C_3_N_4_ quantum dots (Ag–S–gCN QDs), fluorescence quenching upon the addition of Hg^2+^ ions originates from interactions between the fluorophore and the quencher. Time-resolved photoluminescence decay studies were carried out at varying Hg^2+^ concentrations, and the decay profiles were fitted using a biexponential model to determine the average lifetime (*τ*_avg_). An average lifetime of 7.79 ns was exhibited by Ag–S–gCN QDs alone, and with the addition of Hg^2+^ (0.001, 0.01, and 0.4 µM), the lifetimes decreased slightly (7.77, 7.76, and 7.73 ns), correspondingly. This negligible change in lifetime analysis confirmed a static quenching mechanism.^76^

In nanostructured g-C_3_N_4_ systems, static quenching was observed due to ground-state complex formation; at the same time, excited-state electron transfer from g-C_3_N_4_ to Hg^2+^ introduced a dynamic quenching component. Additionally, g-CNQDs fluorescence lifetime measurements show no significant variation upon Hg^2+^ addition, further supporting the dominance of static quenching.^75^ Conversely, in some hybrid systems, dynamic quenching becomes prominent. For instance, in NH_2_-UiO-66/g-CNQDs, the fluorescence lifetime decreases significantly from 25.75 ns to 5.44 ns after the addition of Hg^2+^ ions, indicating dynamic quenching.^77^ Similarly, in CNNPs-CdTe_0_._16_S_0_._84_ quantum dots, dynamic fluorescence quenching for Hg^2+^ was studied with the help of Stern–Volmer plots. The *K*_sv_ values for all ions were significantly lower than 2.61 × 10^9^ M^−1^, which confirmed that the quenching mechanism in this system was governed by dynamic (collisional) processes.^8^

Similarly, carbon nitride nanoparticles were synthesized using ammonium citrate as both the nitrogen source and structural scaffold *via* a solid-state reaction. The resulting NPs were water-soluble and exhibited relatively high quantum yields of 27%. The sensing performance was evaluated against mercury ions and bisphenol-A through fluorescence modulation. The Hg^2+^ induced a strong fluorescence quenching effect with an LOD of 60 nM, whereas bisphenol-A was detected *via* an “off–on” mechanism with an LOD of 45 nM. Importantly, the sensor demonstrated highly selectivity compared to tested mostly heavy metal ions. Most of the metals caused negligible changes, except for Fe^3+^, which only triggered a slight decrease in intensity that was only evident with higher than 600 µM iron concentrations. A particular sharp fluorescence decrease was observed with Hg^2+^, yielding a practical LOD of 0.06 µM. The probe also proved effective in river water samples, showing satisfactory recovery, thereby validating its environmental applicability.^59^

### 3.2 g-C_3_N_4_@metal nanoparticles sensor for mercury sensing

**Scheme 5:**
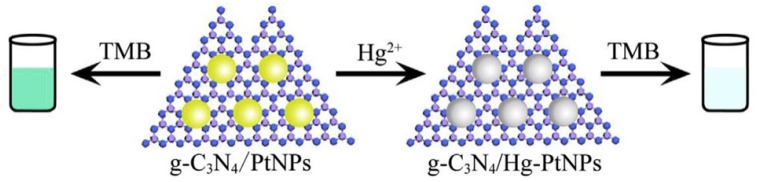
Schematic of g-C_3_N_4_/PtNPs as a colorimetric sensor for Hg^2+^ detection. This figure has been adapted from ref. 78 with permission from Elsevier, copyright 2017.

The decoration of g-C_3_N_4_ with metal nanoparticles provides an effective hybrid material for mercury sensing, enabling amplified signal transduction through improved electron mobility, catalytic surface activity, and strong affinity of mercury toward metallic active sites. For instance, Pt NPs supported on g-C_3_N_4_ nanocomposites (g-C_3_N_4_/PtNPs), synthesized *via* an ultrasonic-assisted process, demonstrated superior oxidase-like catalytic activity for the oxidation of 3,3′,5,5′-tetramethylbenzidine (TMB) compared to that of g-C_3_N_4_ or PtNPs alone. The unique sensing mechanism depicted in Scheme 5 relies on inhibition of the catalytic activity of g-C_3_N_4_/PtNPs upon Hg^2+^ binding, where amalgam formation between Hg and Pt suppresses electron transfer, thereby enabling highly selective colorimetric detection. Notably, the assay showed high selectivity toward Hg^2+^ compared with other metal ions, with an LOD of 1.23 nM. Further extensions to the potential applications in environmental and clinical diagnosis include using g-C_3_N_4_-based hybrid materials as an efficient biocatalyst for environmental monitoring and clinical diagnostics. Nevertheless, the reliance on noble metals, such as Pt, raises concerns regarding cost-effectiveness and large-scale applicability, and the long-term stability of the amalgam-based inhibition mechanism remains unexplored. Despite these challenges, this work illustrates how g-C_3_N_4_-based hybrid catalysts can bridge enzymatic mimics and colorimetric assays, expanding their utility in real-world Hg^2+^ sensing. In the g-C_3_N_4_@metal NP composites, the bridge effect describes how g-C_3_N_4_ supports the metallic active sites *via* electron mobility during amalgam formation with mercury. For instance, in this system, the N of g-C_3_N_4_ is attributed as a bridge for the specific Hg–Pt interaction to form an amalgam. When the concentration of Hg^2+^ increases to 400 nM, the absorption signal of the composite decreased dramatically, indicating inhibition of the catalytic activity of g-C_3_N_4_/PtNPs by converting Hg^2+^ to Hg^0^, thereby forming the PtHg amalgam on the surface of g-C_3_N_4_/PtNPs and inhibiting the catalytic activity.^78^

**Scheme 6:**
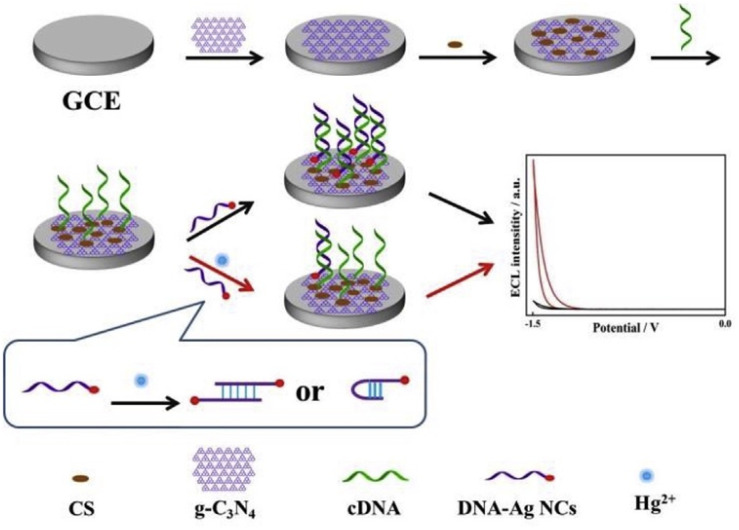
Schematic of Hg^2+^ detection *via* resonance energy transfer between DNA-Ag NCs and g-C_3_N_4_ NSs. This figure has been adapted from ref. 79 with permission from Elsevier, copyright 2019.

Cao *et al.* established an electrochemiluminescence (ECL) platform for Hg^2+^ detection based on the T-Hg^2+^-T coordination chemistry of DNA-Ag NCs and the resonance energy transfer between DNA-Ag NCs and g-C_3_N_4_ NSs, as illustrated in Scheme 6. In this system, g-C_3_N_4_ NSs acted as a stable luminophore, while DNA-Ag-NCs served as a dynamic quencher, enabling a reversible “on–off–on” signal response upon Hg^2+^ binding. This biosensor achieved a LOD of 5 pM over a broad linear range of 0.01–600 nM and demonstrated excellent recovery values (98–116%) with an RSD range of 2.3–4.0%. The proposed ECL sensing platform was utilized in diverse environmental water samples, including Runxi Lake, Ganjiang River, and tap water for Hg^2+^ monitoring. These results underscore the power of integrating DNA nanostructures with g-C_3_N_4_ for constructing highly sensitive ECL biosensors. However, the relatively complex electrode fabrication steps, reliance on costly DNA probes, and potential issues with probe stability may restrict the scalability for routine monitoring. Nonetheless, this work provides a compelling proof-of-concept for harnessing g-C_3_N_4_-based hybrid platforms for environmental mercury detection.^79^ In the case of DNA-based systems that have degradation problems, some practices could improve their stability, including storage at 4 °C in buffer, avoiding freeze-thaw cycles, and protection from light and nuclease contamination.^80,81^

**Scheme 7:**
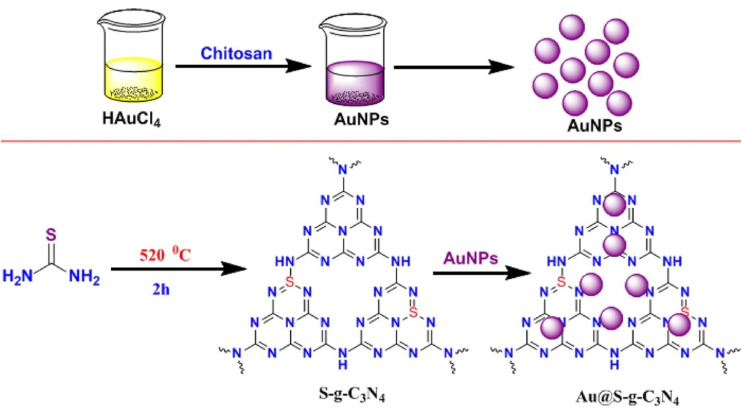
Schematic of the preparation of Au@S–g-C_3_N_4_. This figure has been adapted from ref. 82 with permission from Elsevier, copyright 2018.

Similarly, gold nanoparticles were assembled onto sulfur-doped graphitic carbon nitride to form an Au@S–g-C_3_N_4_ nanocomposite, which was developed for colorimetric detection of mercury ions, where chitosan serves as a green reducing and stabilizing agent (Scheme 7). The resulting nanocomposite was well characterized by FT-IR, UV-Vis, XRD, HR-TEM, Raman, and EDX analyses. In the sensing studies, after the addition of mercury, the formation of Au–Hg amalgam induced a distinct blue shift in the plasmon resonance, causing a noticeable colorimetric change (wine-red to colorless). Au@S–g-C_3_N_4_ attained an LOD of 0.275 nM and good selectivity and sensitivity in the presence of significant metal ions such as alkali, alkaline earth and transition-metal ions, with successful application in spiked river water samples. While the approach demonstrates impressive sensitivity and sustainability, its narrow linear range (100–500 nM) and unaddressed stability under real environmental matrices limit broader applicability.^82^

**Scheme 8:**
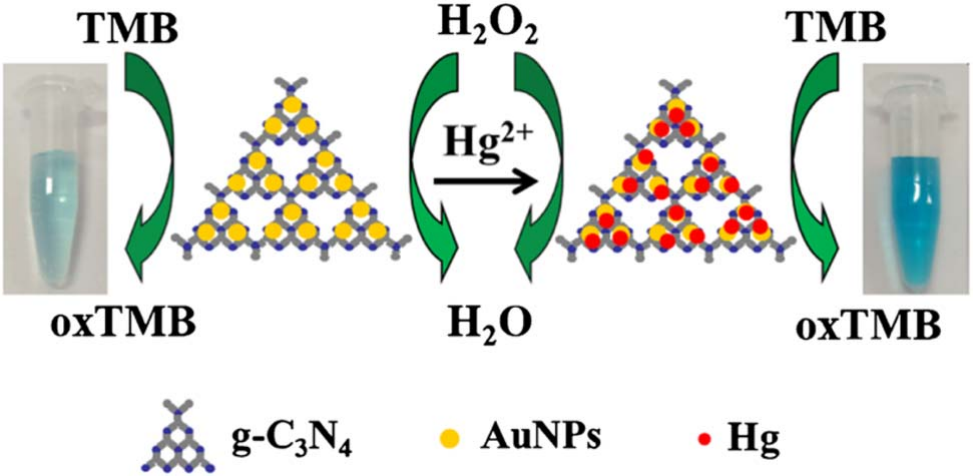
Schematic of the colorimetric detection of Hg^2+^ using g-C_3_N_4_–Au.

In a comparable approach, ultrathin g-C_3_N_4_ nanosheets were employed as a platform for the development of graphitic carbon nitride–gold (g-C_3_N_4_–Au) nanocomposites for the colorimetric and optical detection of Hg^2+^*via* peroxidase-mimicking activity. In the presence of Hg^2+^, catalytic oxidation of 3,3′,5,5′-tetramethylbenzidine by H_2_O_2_ was markedly enhanced with the formation of a blue-colored product, attributed to the *in situ* reduction of Hg^2+^ to Hg^0^ on the g-C_3_N_4_–Au surface, as depicted in Scheme 8. This led to a visible colorimetric change, and the absorption signal (652 nm) increased linearly with Hg(ii) concentration in the range from 5 to 500 nM, achieving an LOD of 3.0 nM. Further, the morphological change of the g-C_3_N_4_–Au nanocomposites in the presence of Hg^2+^ indicates that the nanoparticles aggregate and form larger spherical NPs compared to the as-prepared material. Importantly, the assay exhibits excellent selectivity towards Hg^2+^ ions over other competing metal ions and demonstrates good feasibility for the colorimetric detection of Hg^2+^ ions in real samples, including wastewater, river water, apple juice and orange juice. However, although the platform is cost-effective and offers fast visual detection response, the reliance on citric acid as a reducing agent and the potential influence of natural reductants in real matrices may limit robustness under field conditions.^83^

Similarly, Mahmoudian *et al.* developed Pt/g-C_3_N_4_/polythiophene (Pt/g-C_3_N_4_/PTh NCs) using l-cysteine as a green reductant for Pt^2+^. The hybrid design addresses the low conductivity of pristine g-C_3_N_4_ by incorporating conducting PTh and catalytic Pt NPs, which together enhanced electroactive sites for Hg^2+^ detection. The results revealed that the Pt/g-C_3_N_4_/PTh modified glassy carbon electrode exhibited a marked enhancement in electrocatalytic performance toward the electro-oxidation of 10 µM Hg^2+^ at pH 7 in a mixed electrolyte comprising 0.5 M NaCl, 0.33 M PBS, and 0.1 M LiClO_4_. Remarkably, the sensors achieved an LOD of 0.009 nM and sensitivity of 1.0787 µA nM^−1^ cm^−2^, making it one of the most sensitive electrochemical sensing platforms reported. However, the reliance on costly Pt raises concerns about scalability, and the stability of the composite under long-term real-sample exposure remains unexplored. Despite these drawbacks, the work demonstrates a promising route for combining green chemistry synthesis with multifunctional nanocomposites for ultra-trace Hg^2+^ sensing.^84^ Further, in a study, AgNPs@g-C_3_N_4_ was prepared *via* an *in situ* tannic acid reduction route, providing a green synthesis strategy. Spectroscopic and morphological characterization confirmed the uniform immobilization of AgNPs on g-C_3_N_4_ nanosheets, as evidenced by the surface features of g-C_3_N_4_ and Ag NPs@g-C_3_N_4_. FESEM image analysis of g-C_3_N_4_ revealed non-transparent, layered, sheet-like structures resulting from the aggregation of multiple nanosheets. In contrast, AgNPs@g-C_3_N_4_ composite displayed consistently distributed spherical NPs anchored on the g-C_3_N_4_ sheets. The results verify effective immobilization of AgNPs onto the g-C_3_N_4_ surface, with the nanoparticles exhibiting sizes in the 30–50 nm range. The hybrid exhibited strong SERS sensitivity, enabling detection of methylene blue (MB) and 4-aminothiophenol; more importantly, the quenching of MB SERS signal was utilized for the sensitive detection and quantitative determination of free Hg^2+^ ions in aqueous solution. The platform showed excellent anti-interference ability against Ca^2+^, Mg^2+^, Cu^2+^ and Cd^2+^ ions. While this study highlights a promising SERS-based approach for Hg^2+^ quantification, the reproducibility and scalability of SERS substrates remain challenges, and detection limits must be benchmarked against electrochemical or fluorescence-based systems for a clearer performance comparison.^85^ Similarly, a graphitic carbon nitride-based sensor, Au–g-C_3_N_4_ nanosheets, was developed for Hg^2+^ detection, serving as an efficient on-electrode cathodic co-reactant for Ru(bpy)_3_^2+^ and forming a novel, eco-friendly ECL system. The small Au nanoparticles on the g-C_3_N_4_ nanosheet surface played a crucial role in modulating both cathodic and anodic signals due to their outstanding catalytic activity. Moreover, using Ru(bpy)_3_^2+^ as the sole emitter, with Au–g-C_3_N_4_ nanosheets as the on-electrode co-reactant, enabled rapid ratiometric monitoring of Hg^2+^, supporting the development of simple and fast analytical platforms for mercury determination.^86^

Pd nanoparticles (PdNPs) supported on g-C_3_N_4_ were integrated onto a glassy carbon (GC) electrode for DPV-based Hg^2+^ detection, combining the high surface area of g-C_3_N_4_ with the fast electron-transfer capability of Pd. Morphological analysis confirmed uniform dispersion of PdNPs on the g-C_3_N_4_ surface. Leveraging the synergistic effect of the large surface area of g-C_3_N_4_ nanosheets and the rapid electron-transfer kinetics of PdNPs, the PdNPs/g-C_3_N_4_ GC electrode exhibited excellent electrochemical performance for trace Hg^2+^ detection, with a low LOD of 0.009 µg L^−1^, well below the WHO permissible limit of 1.0 µg L^−1^ and exhibiting a wide linear range of 0.01–15 µg L^−1^. Furthermore, the electrode demonstrated outstanding selectivity, anti-interference capability, and long-term stability. Compared to Au- and Pt-based systems, this PdNPs/g-C_3_N_4_/GC electrode demonstrates superior sensitivity; however, the reliance on Pd, a relatively expensive metal, could hinder large-scale applications. Moreover, this composite underscores the potential application in trace monitoring of Hg^2+^ in real environments, such as tap, local lake, and river water samples.^87^ Similarly, Pt/g-C_3_N_4_/polyaniline nanocomposites synthesized *via* the l-cysteine-assisted reduction of Pt(II), illustrate how the conductivity limitations of g-C_3_N_4_ can be effectively addressed by incorporating conductive polymers such as polyaniline. FESEM images confirmed a minimum of pronounced agglomeration of metallic Pt NPs was observed when synthesized in the presence of g-C_3_N_4_. The modification of GCE with Pt/g-C_3_N_4_/PAn NCs led to significantly enhanced electrocatalytic activity toward the electro-oxidation of Hg^2+^. The composite demonstrates remarkably high selectivity and sensitivity for the detection of Hg^2+^ ions. The resulting composite exhibited a remarkably low LOD of 0.014 nM over a broad linear range (1–500 nM). While this approach provides excellent sensitivity and selectivity, its dependency on platinum raises concerns regarding cost-effectiveness and large-scale application. Moreover, the long-term electrochemical stability of polyaniline in variable environmental conditions remains to be systematically assessed.^88^

**Scheme 9:**
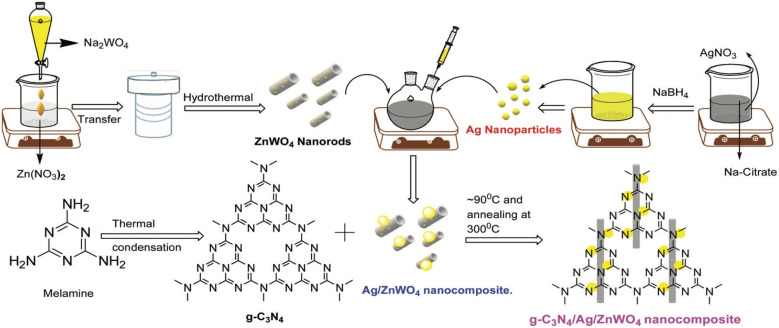
Schematic of the preparation of a g-C_3_N_4_/Ag/ZnWO_4_ nanocomposite. This figure has been adapted from ref. 90 with permission from RSC, copyright 2021.

**Scheme 10:**
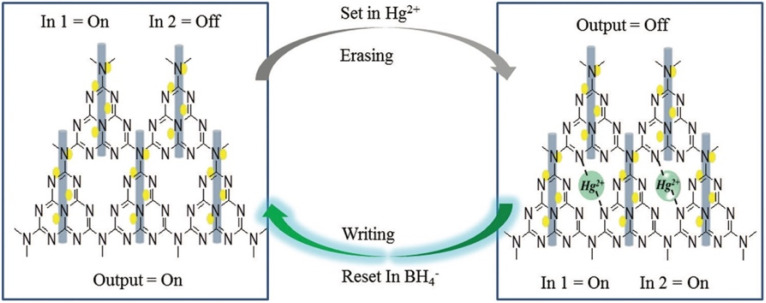
Plausible mechanism for grafting of Hg^2+^ with g-C_3_N_4_/Ag/ZnWO_4_ and further reduction of Hg^2+^ by NaBH_4_. This figure has been adapted from ref. 90 with permission from RSC, copyright 2021.

Chen *et al.* developed an AuNPs/mpg-C_3_N_4_ modified GCE *via* a photochemical route for methylmercury detection. Structural characterization confirmed that Au NPs were dispersed uniformly and anchored on the mpg-C_3_N_4_ surface. The AuNPs/mpg-C_3_N_4_ nanocomposite integrates the excellent catalytic activity of AuNPs with the distinctive porous structure of mpg-C_3_N_4_, leading to enhanced electrochemical sensing performance towards CH_3_Hg^+^. High sensitivity for CH_3_Hg^+^ determination was achieved at the AuNPs/mpg-C_3_N_4_-modified glassy carbon electrode using differential pulse stripping voltammetry. The sensor exhibited good sensitivity (0.285 mA mg^−1^ L^−1^) with an LOD of 0.103 mg L^−1^ in the linear range of 1–25 mg L^−1^ using DPSV. Although the incorporation of two different complexing agents, SnCl_2_ and DTPA, effectively minimized the interference from Hg^2+^, enabling selective CH_3_Hg^+^ detection, the relatively high detection limit compared to natural background levels may restrict its use in environmental monitoring. Nonetheless, testing the CH_3_Hg^+^ concentration of natural waters with a ±106% spike recovery further demonstrates its potential applicability in real sample analysis.^89^ Further in their report, U. K. Ghorui *et al.* designed an Ag-loaded metal tungstate–organic framework nanomaterial (g-C_3_N_4_/Ag/ZnWO_4_) (Scheme 9) that exhibited excellent fluorescence performance towards Hg^2+^ sensing, with a wide linear detection range (0–2 mM) and ultra-low LOD of 0.23 nM in phosphate buffer (pH 7.2). Mechanistic pathway studies revealed that the quenching originated mainly from the embedding of Hg^2+^ onto the nitrogen atoms of g-C_3_N_4_, suggesting a static quenching process, while the material also displayed room-temperature phosphorescence features (Scheme 10). Moreover, the research group extended the work towards molecular information systems by constructing a binary logic function using the response profile of emission intensities and wavelength towards Hg^2+^ ions. So, in the work, the g-C_3_N_4_/Ag/ZnWO_4_ nanocomposite acted as a scavenger for Hg^2+^ ions. Furthermore, the synthesized nanocomposites were successfully applied to the detection of Hg^2+^ ion in selected real samples, including pond water and sewage water, signifying their potential utility in routine Hg^2+^ analysis.^90^

**Fig. 10:**
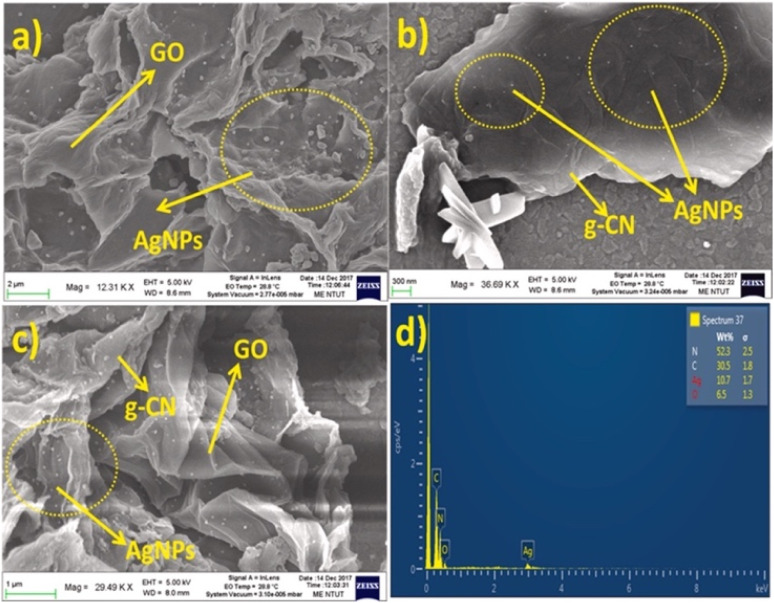
SEM images of (a) AgNPs@GO, (b) AgNPs@g-CN, (c) AgNPs/GO/g-CN and (d) EDAX spectrum of AgNPs/GO/g-CN. This figure has been adapted from ref. 91 with permission from Elsevier, copyright 2021.

In a comparable approach, AgNPs/GO/g-CN nanohybrids were constructed by integrating Ag NPs with graphene oxide and g-C_3_N_4_ to exploit synergistic charge transfer and surface-enhanced Raman scattering properties. The morphology analysis (Fig. 10) revealed that 2D GO and g-C_3_N_4_ sheets provided effective anchoring sites for the AgNPs, which enhanced SERS activity towards methylene blue. The sensing relied on competitive interaction: MB adsorbed on AgNPs *via* its sulfur site to generate strong SERS signals, but, upon addition of Hg^2+^, the metal ion displaced MB from the AgNP surface, leading to a gradual quenching of SERS intensity at diagnostic peaks (≈450, 1400, and 1624 cm^−1^). This displacement-based mechanism enabled quantitative Hg^2+^ detection with a LOD of 0.0199 ppm. These results clearly confirm the interaction between Hg^2+^ ions and methylene blue attached to the surface of the SERS substrate. The influence of mercury concentration was systematically examined by increasing the volume from 10 to 180 µL, with the SERS spectrum recorded at each stage. A gradual decrease in the intensity of the Raman band was observed with increasing Hg^2+^ content. The strong affinity of MB towards metal NPs to the SERS substrate enabled its use as a Raman tag to study selective SERS-based detection of Hg^2+^ ions at an ultra-low concentration of 0.01986 ppm. But MB adsorption and competitive binding raise questions about selectivity in complex wastewater matrices where other thiophilic species (*e.g.*, Pb^2+^, Cd^2+^, and S^2−^) could interfere. Nonetheless, the present SERS substrate is anticipated to be a promising substitute for the determination of wastewater contaminants.^91^

Further, during the development of a sensor for mercury, a photoelectrochemical (PEC) biosensor based on Au NPs-decorated g-C_3_N_4_ (Au NPs@g-C_3_N_4_) was developed by Li *et al.*, who employed the strong photocurrent generation of the composite and the high specificity of thymine-Hg^2+^-thymine (T-Hg^2+^-T) coordination chemistry. The AuNPs enhanced electron–hole separation in g-C_3_N_4_, improving the photocurrent response, while thymine-rich DNA strands immobilized on the electrode selectively captured Hg^2+^ ions. Binding of Hg^2+^ induced T-Hg^2+^-T complexation, which significantly quenched the photocurrent, allowing sensitive PEC detection. The sensor demonstrated an ultra-low LOD of 0.33 pM and a broad linear range (1 pM–1000 nM). Importantly, Au NPs@g-C_3_N_4_ provides a promising platform for the detection of Hg^2+^ and other toxic metal ions in the sub-picomolar range.^19^

### 3.3 g-C_3_N_4_@metal oxide sensors for mercury sensing

**Scheme 11:**
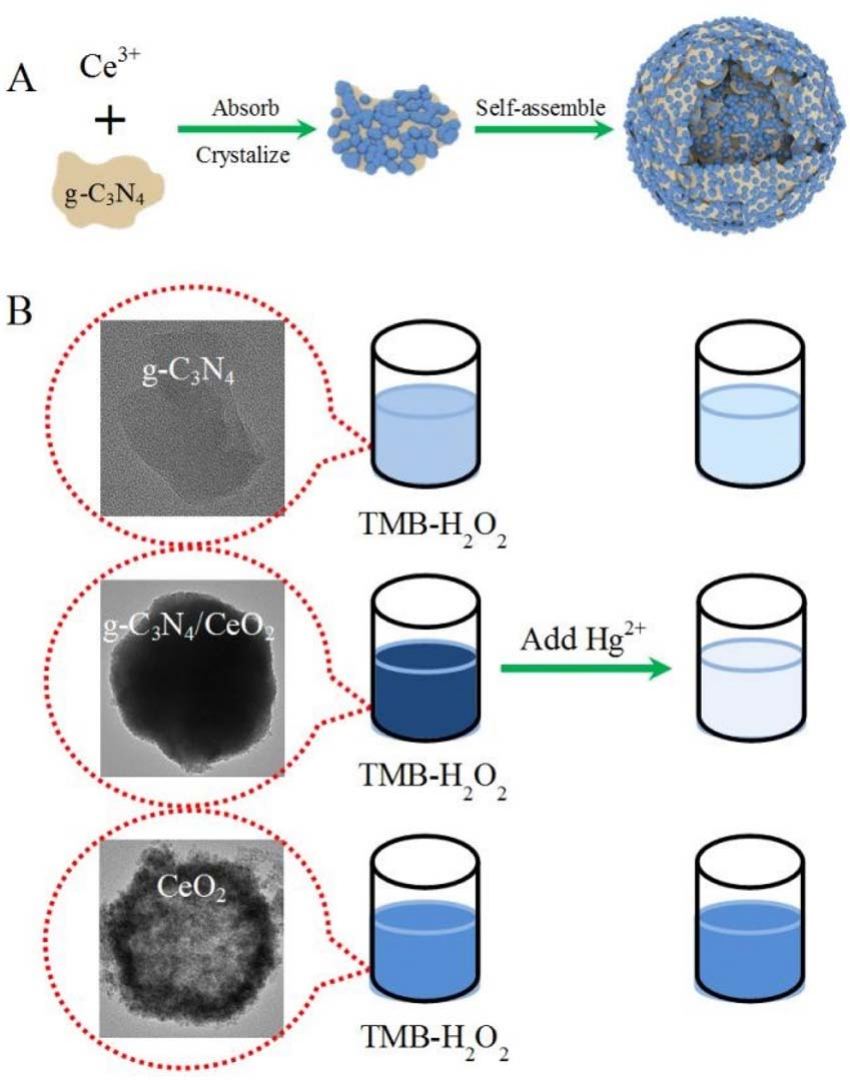
Schematic of (A) the preparation of g-C_3_N_4_/CeO_2_ and (B) colorimetric observation with Hg^2+^. This figure has been adapted from ref. 92 with permission from RSC, copyright 2020.

**Fig. 11:**
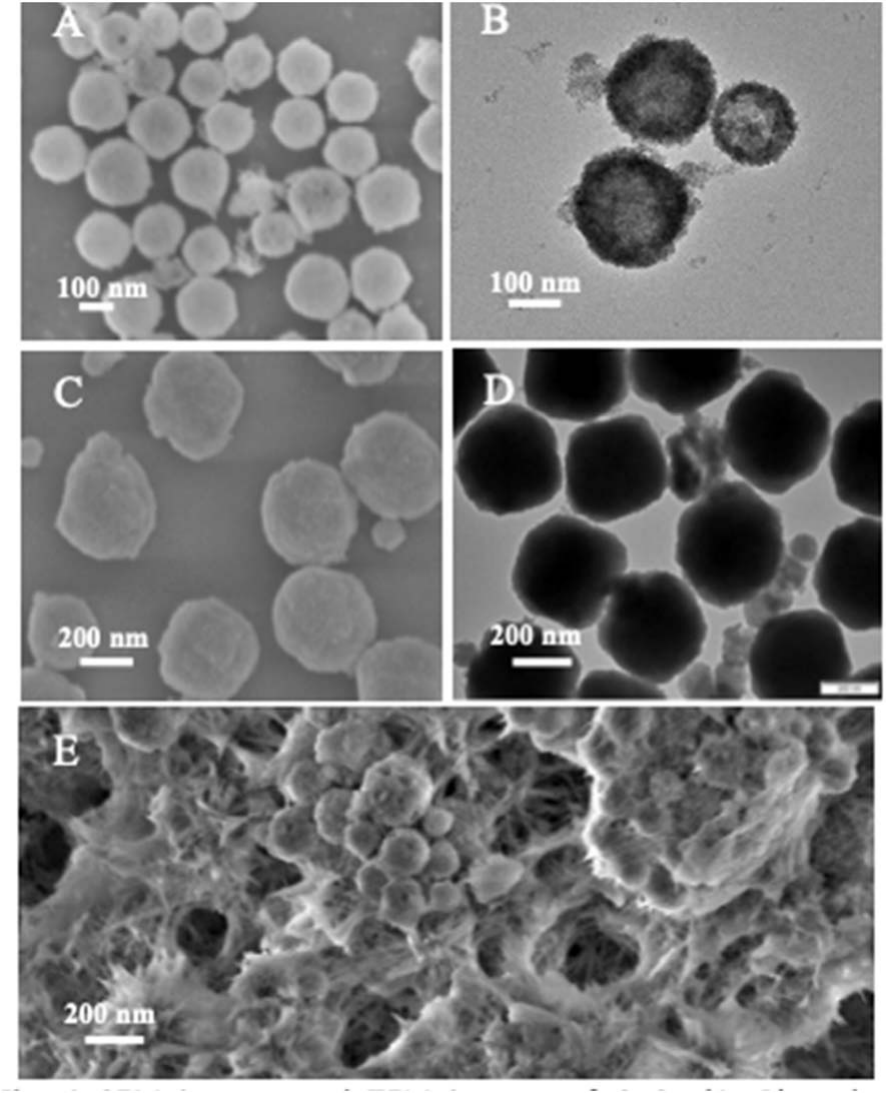
SEM and TEM images of (A and B) CeO_2_ and (C and D) g-C_3_N_4_@CeO_2_ nanocomposite, and (E) SEM image of g-C_3_N_4_@CeO_2_ in the presence of Hg(ii) ions. This figure has been adapted from ref. 92 with permission from RSC, copyright 2020.

The integration of metal oxides with g-C_3_N_4_ signifies a rational materials-engineering approach for mercury sensing, where enhanced surface reactivity, improved charge separation, and strong Hg–O/N interactions collectively contribute to superior sensing performance. For instance, a catalytic sensing strategy based on g-C_3_N_4_/CeO_2_ nanozymes was reported by X. Zhao *et al.* for mercury detection. The hybrid was synthesized *via* a one-pot hydrothermal route in which growing CeO_2_ was formed *in situ* on the g-C_3_N_4_ surface. As illustrated in Scheme 11, Ce^3+^ first undergoes adsorption on the surface of the g-C_3_N_4_ nanosheets to form precursor NPs, assisted by surfactants, resulting in the formation of spherical nanocomposite structures of g-C_3_N_4_/CeO_2_. The morphology analysis of the composite confirmed uniformly monodispersed hollow nanospheres (∼200 nm). The catalytic sensing strategy of g-C_3_N_4_/CeO_2_ for Hg^2+^ relied on significantly suppressed activity of the nanoenzyme upon exposure to mercury due to coordination between Hg^2+^ and the nitrogen of g-C_3_N_4_, as depicted in Fig. 11, which induces nanoparticle aggregation and reduces the surface area. This phenomenon formed the basis of a colorimetric sensing platform for the detection of Hg^2+^ ions selectively, in complex blood and wastewater samples, achieving an LOD of 0.23 nM over a wide concentration range (0.50 nM–800 nM). Importantly, the sensor performed consistently for the colorimetric detection of Hg^2+^ ions in complex samples, including spiked wastewater and blood samples. The measured concentrations of Hg^2+^ ions were found to be in good agreement with the spiked values, yielding satisfactory recovery rates in the range of approximately 94.6–96.8% for wastewater samples. However, the dependence on aggregation effects may limit reusability and reproducibility, suggesting the need for further design optimization in future nanozyme-based sensors.^92^

**Fig. 12:**
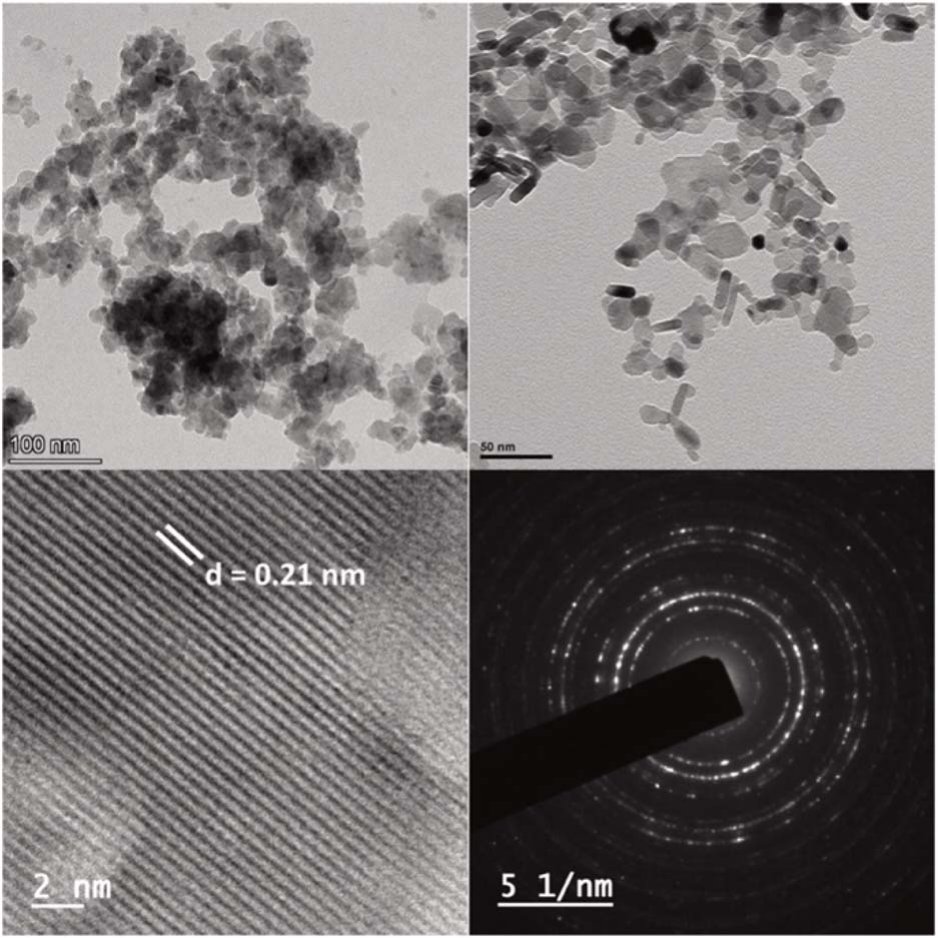
TEM images of NiFe_2_O_4_/g-C_3_N_4_ and SAED pattern of NiFe_2_O_4_/g-C_3_N_4_. This figure has been adapted from ref. 94 with permission from Elsevier, copyright 2023.

**Scheme 12:**
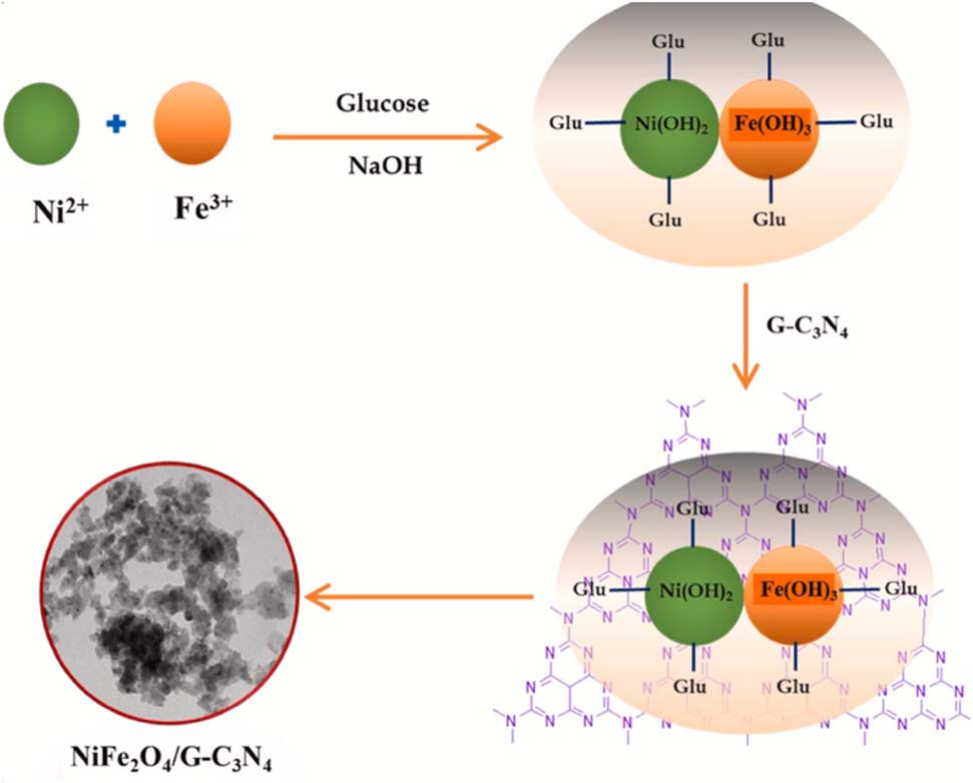
Schematic of the preparation of NiFe_2_O_4_/g-C_3_N_4_. This figure has been adapted from ref. 94 with permission from Elsevier, copyright 2023.

Liu *et al.* reported a Mn_3_O_4_/g-C_3_N_4_ composite with finely dispersed Mn_3_O_4_ NPs enriched with Mn(ii) and Mn(iii), which exhibited superior sensitivity (473.43 µA µM^−1^ cm^−2^) and an ultralow LOD of 0.003 µM for Hg^2+^ compared to Mn_3_O_4_ NPs or g-C_3_N_4_. Mn_3_O_4_ nanoparticles were observed to be uniformly dispersed on the surface of the g-C_3_N_4_, mitigating agglomeration and enhancing conductivity, while g-C_3_N_4_ provided additional adsorption sites to improve Hg(ii) uptake. A heterojunction was formed between the Mn_3_O_4_ and g-C_3_N_4_, facilitating electron transfer from g-C_3_N_4_ to Mn_3_O_4_. This electron flow enables the interconversion of Mn(iv) to Mn(iii) and Mn(ii), promoting the Mn(ii)/Mn(iii)/Mn(iv) redox cycle and thereby enhancing Hg(ii) redox processes. Furthermore, the sensor demonstrated excellent stability, reproducibility, selectivity, and anti-interference capability, and was applied in real water samples. While the report highlights the promise of Mn-based composites, scalability and long-term electrode durability remain areas requiring further exploration.^93^ Further, a pencil graphite electrode modified with NiFe_2_O_4_/g-C_3_N_4_ nanocomposites was developed for Hg^2+^ sensing, wherein bimetallic oxides were heterogeneously grown over the g-C_3_N_4_ sheets *via* a wet chemical approach. TEM and HRTEM images (Fig. 12) confirmed the uniform dispersion of NiFe_2_O_4_ over the g-C_3_N_4_ nanosheets, a modification that enhanced charge transfer kinetics at the electrode–electrolyte interface, which was the key factor responsible for the superior electrochemical performance compared to bare PGE, g-C_3_N_4_/PGE, and NiFe_2_O_4_/PGE. The modified electrode displayed a wide linear detection range of 10–800 nM, with a LOD of 2.49 nM. Although the LOD is not the lowest among the g-C_3_N_4_-based composites, it remains well below the WHO guidelines, supporting its practical applicability. The sensor further demonstrated good reproducibility, stability, and tolerance against interfering ions, and was successfully validated in real water matrices (Scheme 12).^94^

**Scheme 13:**
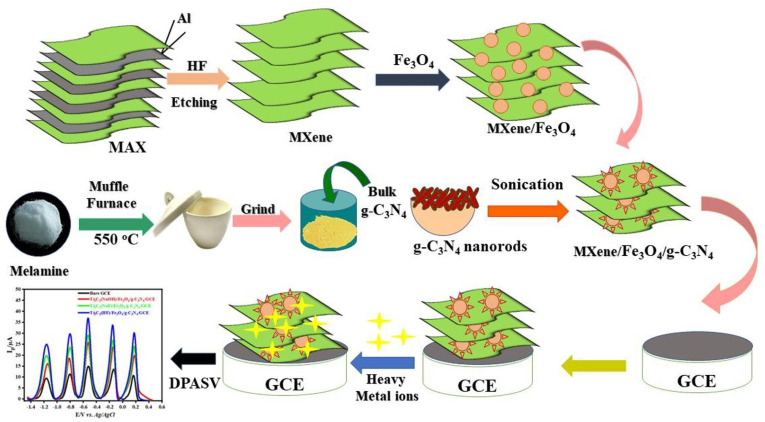
Schematic of the preparation of MXene/Fe_3_O_4_/g-C_3_N_4_. This figure has been adapted from ref. 96 with permission from Elsevier, copyright 2023.

Similarly, it was reported that V_2_O_5_-doped g-C_3_N_4_ was developed for combined electrochemical detection and photocatalytic elimination of Hg^2+^ from aqueous systems. V_2_O_5_/g-C_3_N_4_ nanosheets were synthesized *via* direct thermal decomposition using varying dopant loadings ranging from 0.1 to 0.5 wt%. Notably, the optimized 0.2 wt% V_2_O_5_/gCN demonstrated superior electrochemical sensitivity (9.9857 µA µM^−1^ cm^−2^) and a wide linear detection range (0.2–100 µM), with an LOD of 9.2 nM. Photocatalytic removal under visible light was also enhanced, with near-complete Hg^2+^ elimination in a short treatment time, as confirmed by ICP-OES analysis (2762 ppm → 101 ppm). Therefore, this study provides valuable insights for the rational design of direct Z-scheme photoelectrocatalytic systems with potential applications in real-time sensing and environmental remediation.^95^ In a later study, Akhtar and co-workers reported a g-C_3_N_4_-derived nanocomposite, Ti_3_C_2_/Fe_3_O_4_/g-C_3_N_4_, which enabled the concurrent sensing of Zn^2+^, Cd^2+^, Pb^2+^, Cu^2+^, and Hg^2+^ ions, as illustrated schematically in Scheme 13. The as-prepared nanocomposites were well characterized using standard techniques. Differential pulse anodic stripping was employed for the concurrent determination of Zn^2+^, Cd^2+^, Cu^2+^, Pb^2+^, and Hg^2+^ ions. Key electrochemical parameters, including solution pH, loading of Ti_3_C_2_/Fe_3_O_4_/g-C_3_N_4_ on the glassy carbon electrode (GCE), were examined, and systematic optimization of the supporting electrolyte and deposition time was carried out to achieve enhanced sensitivity. Under the optimized conditions, the Ti_3_C_2_(HF)/Fe_3_O_4_/g-C_3_N_4_-modified electrode exhibited excellent analytical performance toward all five metal ions over a concentration range of 0.005–0.5 µmol L^−1^. The enhanced sensing behavior was attributed to the hetero-structured architecture and the strong coordination capability of MXene and Fe_3_O_4_/g-C_3_N_4_, which collectively promoted efficient interfacial charge transfer and provided abundant adsorption sites for metal ions. The sensor was further validated for metal ion analysis in tap water samples, yielding reliable and satisfactory analytical performance. In addition, the sensing platform demonstrated excellent resistance to interference, a wide linear dynamic range, and low limits of detection. The LODs were determined to be 0.26 nM for Zn^2+^, 0.21 nM for Cd^2+^, 0.10 nM for Pb^2+^, 0.11 nM for Cu^2+^, and 0.12 nM for Hg^2+^, and all values were significantly lower than the guideline limits prescribed by WHO. The favorable recoveries obtained from real water samples further confirm the practical applicability of the developed composite sensor. When benchmarked against earlier g-C_3_N_4_-based composite systems, the Ti_3_C_2_/Fe_3_O_4_/g-C_3_N_4_ system showed notable improvements in sensitivity and multi-ion detection capability, highlighting the potential of MXene-integrated nanocomposites for environmental monitoring. Future studies could focus on long-term stability and large-scale deployment.^96^

**Scheme 14:**
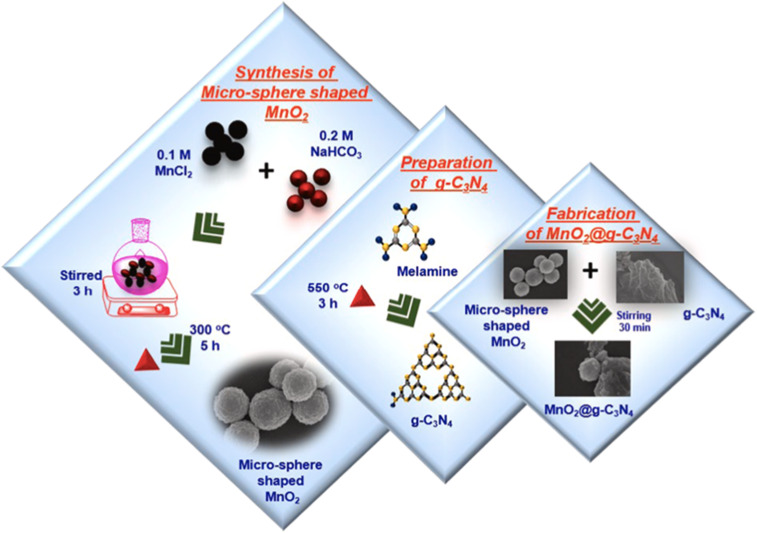
Schematic of synthesis of MnO_2_, g-C_3_N_4_ and the MnO_2_@g-C_3_N_4_ composite. This figure has been adapted from ref. 97 with permission from Elsevier, copyright 2024.

**Fig. 13:**
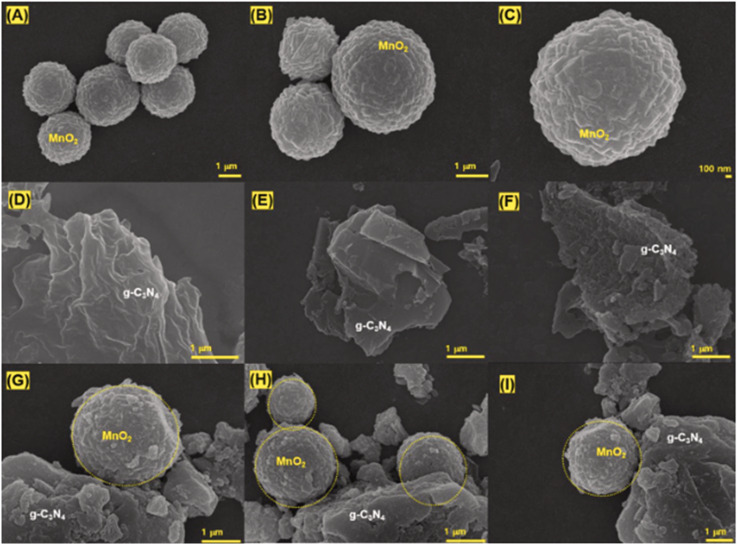
FESEM images at different magnifications of (A–C) MnO_2_, (D–F) g-C_3_N_4_, and (G–I) the MnO_2_@g-C_3_N_4_ composite. This figure has been adapted from ref. 97 with permission from Elsevier, copyright 2024.

Venkatesh and co-workers reported the synthesis of an MnO_2_@g-C_3_N_4_ nanocomposite (Scheme 14) for the electrochemical sensing of Hg^2+^ ions. The material was constructed by combining MnO_2_ with a two-dimensional, nitrogen-rich g-C_3_N_4_ carbon framework, which serves as both a conductive support and active site for heavy metal ion detection. Structural and morphological characterization revealed that the chemically synthesized MnO_2_ exhibited 0D microsphere particles. Upon annealing, melamine was transformed into an asymmetrical, lamellar, and wrinkled sheet-like structure (Fig. 13), which provides a high surface area and abundant active sites. Ultrasonication facilitated uniform deposition of MnO_2_ particles on the uneven g-C_3_N_4_ sheets and formed a MnO_2_@g-C_3_N_4_ composite with abundant electroactive sites. Subsequently, when applied MnO_2_@g-C_3_N_4_ as a composite-modified screen-printed carbon electrode (MnO_2_@g-C_3_N_4_@SPCE) for sensing Hg^2+^, the sensor exhibited an LOD of 2.6 nM over an extensive linear range with excellent selectivity and repeatability. Additionally, the MnO_2_@g-C_3_N_4_ sensor exhibited high recovery rates for Hg^2+^ during real-sample analysis, particularly in river water, underscoring its practical applicability. The improved electrochemical response is attributed to the synergistic interaction between MnO_2_ and g-C_3_N_4_, resulting in abundant electroactive sites and facilitating efficient charge transfer. Consistently superior recovery values further confirmed the practical feasibility of the MnO_2_/g-C_3_N_4_ composite sensor. Overall, the excellent electrochemical response can be ascribed to the combined effect of the metal oxide (MnO_2_) and the carbon-based support (g-C_3_N_4_) when integrated onto a screen-printed carbon electrode (SPCE).^97^

Sairaj *et al.* reported the preparation of a praseodymium oxide-boron co-doped g-C_3_N_4_ composite through a one-step solid-state thermal condensation method, using urea, orthoboric acid, and praseodymium nitrate as precursors. The incorporation of praseodymium and boron into the g-C_3_N_4_ framework was found to enhance its electronic conductivity and create additional active sites, improving its performance in catalytic and sensing applications. The resulting composites were comprehensively characterized using multiple analytical techniques. All samples exhibited multifunctional behavior, demonstrating efficient peroxidase-mimetic activity for the colorimetric detection of Hg^2+^ ions, along with enhanced photocatalytic performance for the degradation of textile dyes, photoreduction of hexavalent chromium, and reduction of nitroaromatic compounds, such as *p*-nitrophenol and *p*-nitroaniline. The improved photocatalytic activity was primarily ascribed to the formation of a type-II heterojunction, which promotes effective charge separation and extends the lifetime of photogenerated charge carriers. Furthermore, the porous flake-like morphology, reduced optical band gap, and the conductive nature of praseodymium oxide collectively contributed to the enhanced catalytic efficiency. For Hg^2+^ detection, a linear range of 0.25–800 nM and a LOD of 42.4 nM were reported using a colorimetric approach. Mechanistically, Hg^2+^ coordination with nitrogen lone pairs in g-C_3_N_4_ induces catalyst aggregation, suppressing catalytic activity in a concentration-dependent manner. Compared with other g-C_3_N_4_-based sensors, this Pr/B co-doped system demonstrates versatile applicability in simultaneous sensing and pollutant degradation, although its LOD is higher than those of electrochemical sensors, suggesting potential for further optimization.^98^

### 3.4 g-C_3_N_4_ QDs sensor for mercury sensing

**Fig. 14:**
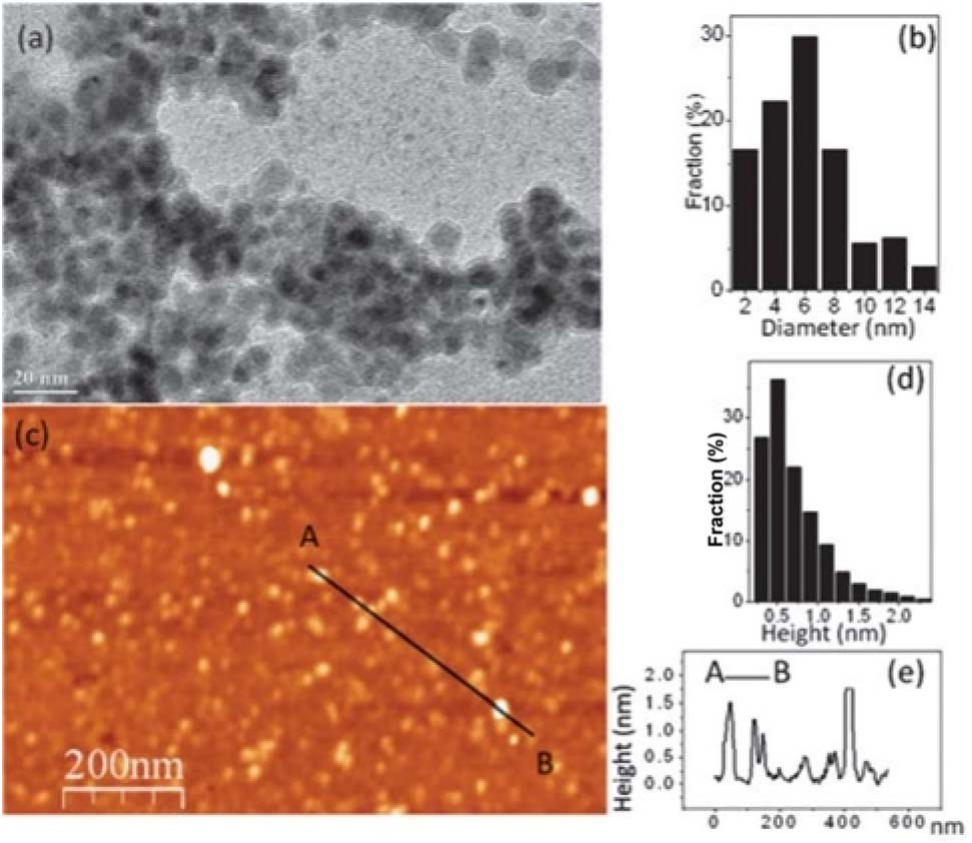
(a) TEM image (b) g-CNQDs diameter distribution, (c) AFM image of g-CNQDs, (d) g-CNQDs height distribution and (e) height profile. This figure has been adapted from ref. 100 with permission from RSC, copyright 2012.

g-C_3_N_4_ QDs are one of the most robust motifs of the carbon family. Since their emergence in 2014,^99^ g-C_3_N_4_ quantum dots (QDs) have attracted considerable attention due to their unique physicochemical characteristics, encompassing structural, morphological, electrochemical, and optoelectronic properties. Graphitic carbon nitride quantum dots (g-C_3_N_4_ QDs) exhibit several advantageous features, including a non-zero band gap, facile chemical functionalization and doping, highly tunable optical properties, excellent dispersibility, and good biocompatibility. These attributes render g-C_3_N_4_ QDs promising candidates for a broad range of applications across sensing, catalysis, and biomedical fields. In comparison with the common carbon-based QDs, g-C_3_N_4_ QDs are endowed with electron-rich features, basic surface moieties, and H-bonding motifs owing to the existing N and H atoms. Hence, they are regarded as interesting candidates to further complement carbon-based QDs in functional materials applications. Because of the prominent quantum confinement and edge effects, the optical and physical characteristics of g-C_3_N_4_ QDs are distinct from those of other members of the carbon materials family. For instance, as Barman and Sadhukhan reported, a microwave-assisted synthesis of fluorescent graphitic carbon nitride quantum dots (g-CNQDs) formamide, yielded few-layer QDs (<2 nm thick) with an average size of 7 nm (for TEM images, see Fig. 14a). AFM images (Fig. 14c) revealed topographic heights of particles (0.3–2.1 nm), suggesting that g-CNQDs consists of either a single layer or a few layers of CNx sheets. The g-CNQDs exhibited a selective fluorescence quenching response in the presence of Hg^2+^, attributed to the strong affinity of Hg^2+^ towards nitrogen atoms and the formation of a stable g-CNDS–Hg^2+^ complex. The g-CNQDs do not exhibit significant spectral change in the presence of alkali metals, other heavy metals, or transition metals, although notable minor spectral changes were observed for Cu^2+^ and Ni^2+^. A LOD of ∼10^−9^ M was achieved for Hg^2+^ ions, significantly lower than conventional dye-quencher systems, highlighting the ‘superquenching’ of fluorescence by g-CNQD and Hg^2+^ duos. The pronounced superquenching of g-CNQDs by Hg^2+^ ions is likely attributed to a static quenching mechanism arising from the formation of a stable, non-fluorescent g-CNQD–Hg^2+^ complex. In addition, the resulting g-CNQD–(Hg^2+^)_*x*_ system functions as a highly selective and sensitive fluorescence OFF–ON sensor for iodide ions in aqueous media. Consequently, these graphitic carbon nitride quantum dots demonstrate significant potential as rapid and highly selective sensing platforms for both Hg^2+^ and iodide ions in biological and environmental samples. While this work demonstrates remarkable sensitivity, some limitations remain. Minor cross-reactivity with Cu^2+^ and Ni^2+^ suggests that matrix interference in real water samples may affect performance.^100^

**Scheme 15:**
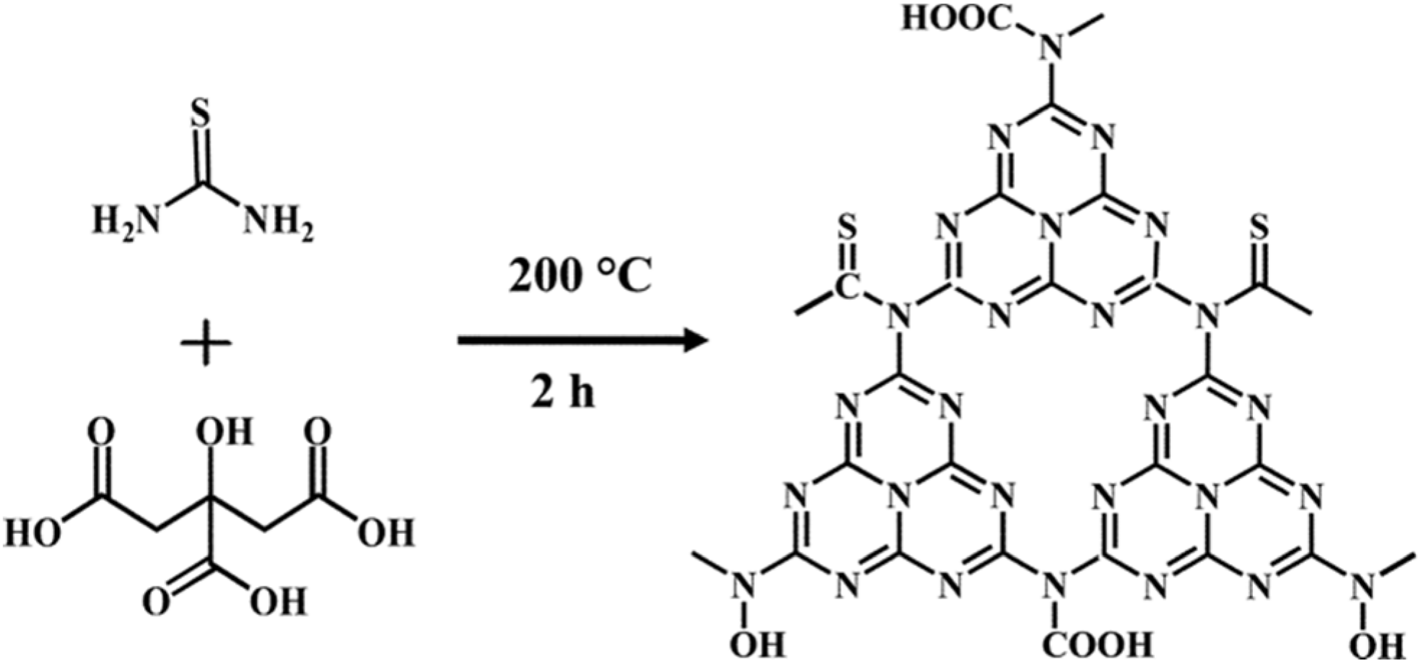
Schematic of the preparation of OS-GNCQDs.

**Fig. 15:**
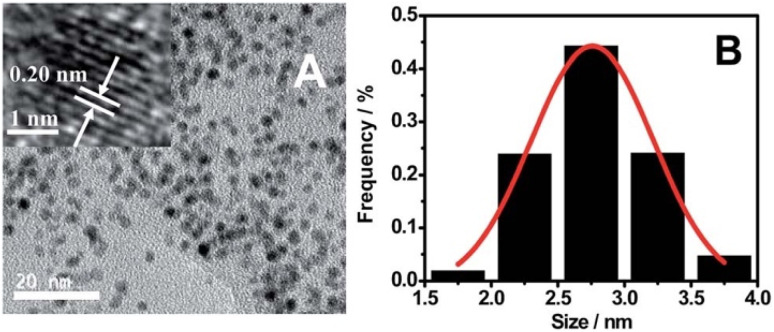
(A) TEM image of OS-GNCQDs (b) size distribution. This figure has been adapted from ref. 101 with permission from RSC, copyright 2015.

In another study, g-C_3_N_4_ QDs were prepared *via* a single-step MW-assisted protocol and characterized by XRD, TEM, FTIR, and XPS, revealing abundant surface hydroxyl and carbonyl groups. Interestingly, instead of conventional fluorescence, a chemiluminescence (CL)-based sensing platform was developed using K_3_Fe(CN)_6_ as an oxidant. The CL emission of the g-CNQDs was efficiently quenched in the presence of Hg^2+^, attributed to non-radiative electron transfer from the excited states of g-CNQDs to the d-orbitals of Hg^2+^ ions. The sensor demonstrated a remarkably low LOD of 0.08 ng mL^−1^ over the linear response range of 0.25–10 ng mL^−1^ and achieved recoveries of ∼96–103% in spiked water and food samples, underlining its practical potential. The established method was successfully employed for the selective and sensitive detection of Hg^2+^ in water and food samples with excellent recoveries for the spiked samples. Although significant progress has been achieved, many challenges still need to be addressed. The reliance on an oxidizing agent (K_3_Fe(CN)_6_) may complicate real-sample analysis, especially in biological contexts where oxidants can interfere with matrix components.^4^ Lu *et al.* prepared oxygen and sulfur co-doped graphitic carbon nitride quantum dots (OS-GCNQDs) *via* thermal treatment of citric acid and thiourea, as shown in Scheme 15. The resulting spherical QDs (2.78 nm) (Fig. 15) exhibited selective sensing of Hg^2+^ ions, whereby the PL intensity of OS-GNCQDs was strongly quenched in the presence of Hg^2+^, even against most competing metal ions. The enhanced selectivity and specificity were attributed to the high affinity of Hg^2+^ towards amino groups and thiourea functionalities present on the surface of OS-GNCQDs, establishing sensitive detection in the range of 0.001–20.0 mM, with a LOD of 0.37 nM. Notably, OS-GCNQDs were further evaluated for cell imaging, exhibiting satisfactory biocompatibility and highlighting their potential as fluorescent probes for biosensing and bioimaging. While the co-doping strategy clearly enhances performance relative to pristine g-CNQDs, critical issues remain. The study did not fully assess possible interference from other soft metal ions (*e.g.*, Ag^+^, Pb^2+^), which could compromise selectivity in real-world conditions.^101^

Similarly, Cao *et al.* synthesized fluorescent carbon nitride quantum dots (CNQDs) within 5 minutes using the oleic acid mediated reaction of citric acid and urea. The as-prepared CNQDs were well characterized *via* standard analytical techniques. The CNQDs displayed excitation-dependent fluorescence with dual emission maxima at 450 and 540 nm. Selective fluorescence quenching was observed for Hg^2+^, with negligible response to other ions. The fluorescence intensity of the CNQDs decreased significantly upon the addition of Hg^2+^, indicating their high selectivity for this ion. Interestingly, concentration-dependent studies revealed two distinct linear regions (0.1–10 µM and 10–30 µM), implying the presence of multiple binding sites for Hg^2+^ on the CNQD surface. Its LOD of 0.14 µM, and its successful application in tap and groundwater, demonstrated its practical feasibility for environmental monitoring. Although this rapid synthesis method is attractive, the detection limit is higher than those of other CNQD-based sensors (often reaching the nM or sub-nM range). Thus, while effective for environmental water monitoring, its sensitivity may be insufficient for the trace-level detection required for biological samples or regulatory standards. The unique dual-emission and dual-binding behavior, however, provides valuable insight into surface-ion interactions and could be exploited in the design of multiplex or ratiometric sensing platforms.^102^

**Scheme 16:**
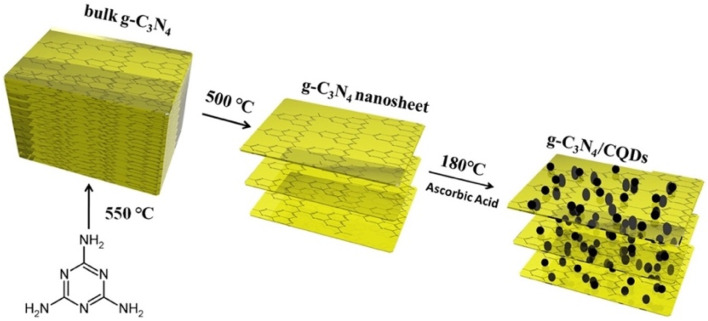
Schematic of the preparation of metal-free g-C_3_N_4_/CQDs composites. This figure has been adapted from ref. 103 with permission from Elsevier, copyright 2017.

**Fig. 16:**
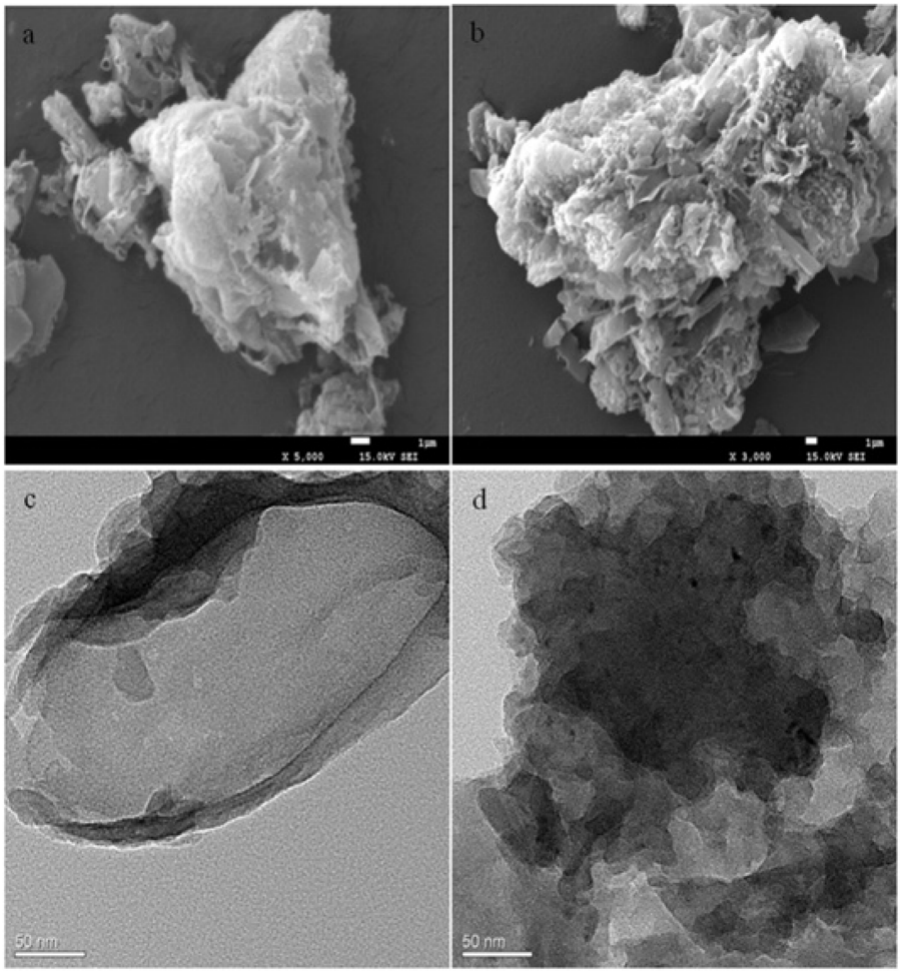
SEM images of (a) pure g-C_3_N_4_ and (b) g-C_3_N_4_/CQDs composites. TEM images of (c) pure g-C_3_N_4_ and (d) g-C_3_N_4_/CQDs composites. This figure has been adapted from ref. 103 with permission from Elsevier, copyright 2017.

Zhou *et al.* explored photochemical vapor generation (PCVG) using a g-C_3_N_4_/carbon quantum dot composite as a metal-free photocatalyst for total mercury detection by ICP-MS. The composite was prepared as shown in Scheme 16 and well characterized *via* different techniques. SEM images (Fig. 16) clearly revealed pure g-C_3_N_4_ and composite morphology and confirmed the successful incorporation of CQDs between the layers of g-C_3_N_4_ sheets. The incorporation of CQDs enhanced visible-light absorption and electron–hole separation, enabling efficient reduction of Hg^2+^ to Hg^0^, while HCOOH served as a hole scavenger. This system achieved an impressive LOD of 8 ng L^−1^, ranking among the most sensitive mercury detection methods. While the work demonstrates the strength of g-C_3_N_4_/CQD composites in green, light-driven mercury detection, practical limitations remain. The method relies on sophisticated ICP-MS instrumentation, which restricts field applicability.^103^

**Scheme 17:**
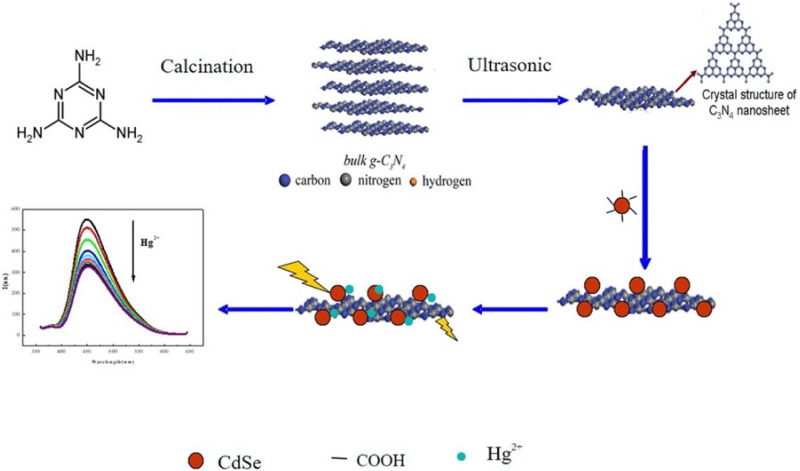
Schematic of the mechanism of FRET-based detection of Hg^2+^. This figure has been adapted from ref. 104 with permission from Springer, copyright 2018.

Similarly, a FRET system has been constructed based on CdSe quantum dots (QDs) (donor) and g-C_3_N_4_ (receptors) for Hg sensing (Scheme 17). The nanocomposites were characterized by XPS, XRD, FTIR and TEM, revealing that the g-C_3_N_4_ nanosheets were decorated randomly by CdSe QDs (∼7 nm). The feasibility of the FRET system as a sensor was demonstrated by Hg(ii) detection in water, wherein the sensor demonstrated fluorescence quenching upon addition of Hg^2+^, with a linear concentration range 0–32 nmol L^−1^, and an LOD of 5.3 nmol L^−1^ at pH 7. The g-C_3_N_4_ nanosheets and CdSe quantum dots exhibited negligible fluorescence quenching in the presence of competing metal ions, apart from Hg^2+^, indicating the high selectivity of the sensing system. The CdSe QDs/g-C_3_N_4_ nanosheet assembly functioned effectively as a FRET-based sensor, providing a sensitive and selective platform for Hg^2+^ detection in real samples. Analysis of well, lake, and tap water samples yielded Hg^2+^ recoveries in the range of 95.4–101.6%. This strategy thus offers an alternative route for the design of FRET-based sensors for Hg^2+^ determination in aqueous environments, with potential applicability in both environmental and biological matrices. However, the random decoration of QDs on g-C_3_N_4_ may affect the reproducibility and long-term stability of the sensing platform. Compared with other g-C_3_N_4_-based fluorescence sensors, the LOD achieved here is competitive but not significantly superior, suggesting that the real merit lies in its integration of QDs for enhanced signal modulation.^104^

**Scheme 18:**
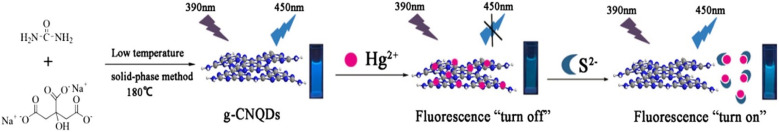
Schematic of the detection of Hg^2+^ and S^2−^. This figure has been adapted from ref. 106 with permission from Springer, copyright 2018.

A facile one-pot thermal synthesis of sulfur- and oxygen-doped carbon nitride quantum dots (SCNQDs) has been reported using thiourea and EDTA disodium salt as precursors. The SCNQDs were characterized by UV-Vis, FT-IR, XPS, powder XRD, and TEM imaging. The SCNQDs demonstrated exceptional sensitivity towards Hg^2+^, showing a linear response between 10 nM and 1 µM, with an impressively low LOD of 0.01 nM. Notably, the sensing performance was consistent across double-distilled and tap water, highlighting strong practical applicability. Extending the platform, SCNQDs were immobilized on filter paper to create a portable solid-phase detection system, which retained comparable sensitivity and selectivity. Furthermore, a ready-to-use detection system was developed by loading SCNQDs onto filter paper, enabling the selective detection of Hg^2+^ in both biological fluids and environmental samples, which was not previously available, according to the researchers' knowledge. From a critical perspective, this work advances the field in two key ways: (i) achieving ultralow LODs compared to other g-C_3_N_4_-based QD sensors, and (ii) validating portability through filter paper integration, a step towards point-of-care applications. However, some limitations are associated with the approach; for instance, operational stability of SCNQDs was not comprehensively addressed under varying pH and ionic strengths, which is crucial for real-world deployment.^105^ Similarly, g-CNDs have also been explored as dual-mode fluorescent probes for the detection of Hg^2+^ and sulfide by Wang *et al.* The probe exhibited a fluorescent turn-off upon interaction with Hg^2+^; Hg^2+^ binding induced strong fluorescence quenching of the blue g-CNQD fluorescence over a linear response of 0.20 to 21 µM, with a LOD of 3.3 nM. Subsequent sulfide addition restored the fluorescence signal over the linear response 8.0 to 45 µM, with a LOD of 22 nM. The mechanism of the “turn-off–on” is illustrated in Scheme 18. The established protocol was assessed using spiked tap water, lake water, and wastewater samples, to which Hg^2+^ concentrations of 1.00, 9.00, and 18.00 µmol L^−1^ were added. The obtained recoveries ranged from 99.0% to 110.0%, demonstrating satisfactory analytical performance for Hg^2+^ determination in diverse water matrices. These results further support the potential of g-CNQDs as reliable probes for Hg^2+^ detection in real environmental samples. From a critical standpoint, this work stands out for introducing a reversible “turn-off/on” sensing system, extending beyond single-analyte detection. Such dual recognition is highly relevant for environmental monitoring, where coexisting ions can complicate selectivity.^106^

**Fig. 17:**
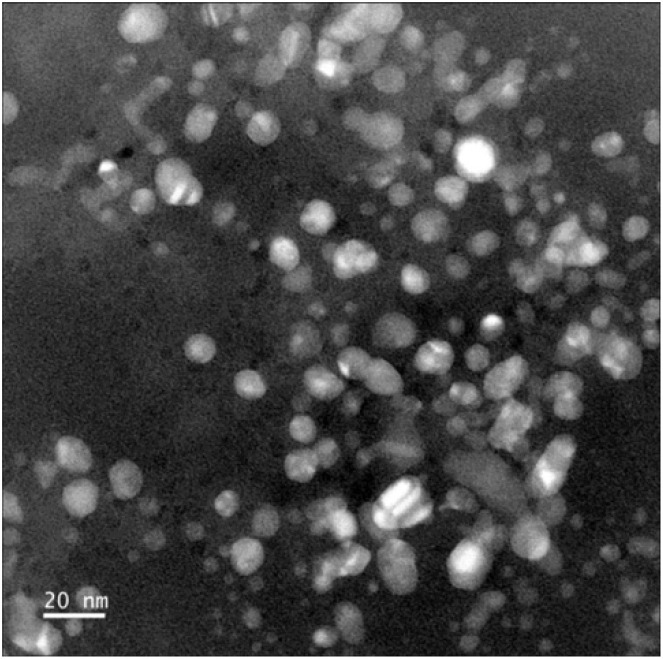
TEM image of T-gCNQDs at (20 µm scale bar). This figure has been adapted from ref. 107 with permission from Springer, copyright 2018.

**Scheme 19:**
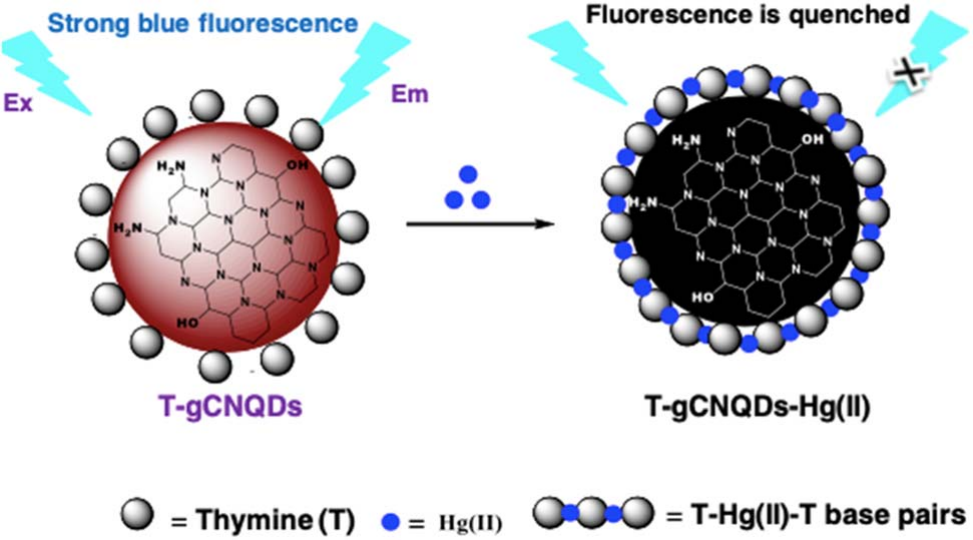
Schematic of Hg^2+^ detection by T-gCNQDs *via* fluorescence quenching. This figure has been adapted from ref. 107 with permission from Springer, copyright 2018.

Owing to the specific interaction between thymine and Hg^2+^, a thymine-regulated gCNQDs (T-gCNQDs) composite was synthesized *via* a facile microwave-assisted hydrothermal route. The incorporation of thymine enhances the photoluminescence properties of gCNQDs, and TEM analysis (Fig. 17) revealed quasi-spherical and monodispersed particles (3–8 nm). Optical studies reveal a strong absorption peak at 271 nm, which is attributed to the pi–pi* transition of the C–N rich heptazine unit in the T-gCNQDs. Fluorescence at 350/445 nm was selectively quenched by Hg(ii) compared to the thymine-free nanoprobe. Notably, the probe achieved an impressive LOD of 0.15 nM over a linear range of 1.0 to 500 nM. Mechanistic studies revealed that quenching, attributed to static complex formation (T-Hg(ii)-T), reflects the strong binding affinity of Hg(ii) to the surface thymine moieties, as depicted in Scheme 19. The quenching efficiency for the T-gCNQDs–Hg(ii) system was observed to be enhanced ∼5 to 6 orders of magnitude compared with thymine-free gCNQDs. This profound enhancement can be attributed to the introduction of thymine moieties onto the gCNQDs surface, which significantly influences the extent of fluorescence quenching through either direct coordination with Hg(ii) ions and/or Hg(ii)-induced aggregation of T-gCNQDs. Furthermore, the interaction between Hg(ii) and T-gCNQDs is likely to suppress interfacial electron–hole (radiative) recombination processes, thereby resulting in efficient fluorescence quenching. Further, the sensor demonstrated excellent recoveries in real water samples collected from the tap and a pond. But the report did not address potential interference from natural organic matrices, which could limit environmental applicability.^107^

**Scheme 20:**
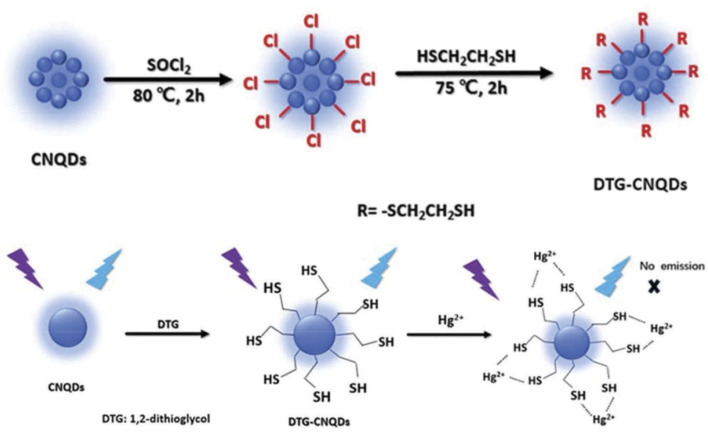
Schematic of the synthesis of DTG-CNQDs and fluorescence quenching by Hg^2+^.

In another report, 1,2-dithioglycol (DTG) functionalized carbon nitride quantum dots (DTG-CNQDs) were fabricated *via* surface modification, yielding strong blue fluorescence under ultraviolet light and with a high quantum yield of 27%. DTG-CNQDs displayed selective quenching towards Hg^2+^ in phosphate buffer (pH 6.0) due to strong affinity between them (Scheme 20), with a linear response range of 0.020–0.50 µM and an LOD of 0.63 nM. Mechanistic investigations indicated two plausible fluorescence quenching pathways. First, Hg^2+^ ions exhibit strong coordination affinity toward thiol groups; upon binding to DTG-CNQDs *via* thiol functionalities, the photogenerated electrons within the DTG-CNQDs are readily captured by Hg^2+^ under ultraviolet irradiation. This photoinduced electron-transfer process effectively suppresses radiative recombination, leading to fluorescence quenching. Second, aggregate-induced quenching, confirmed by UV-Vis and TEM evidence of Hg^2+^ induced agglomeration of DTG-CNQDs. Furthermore, the sensor was successfully employed for the detection of Hg^2+^ in real water samples, demonstrating its broad potential application in environmental monitoring and analytical assessment. Despite these advantages, the linear range remains narrower than those of other CNQD-based probes, and possible interference from competing thiophilic ions, such as Ag^+^ or Pb^2+^, was not systematically evaluated.^67^

**Scheme 21:**
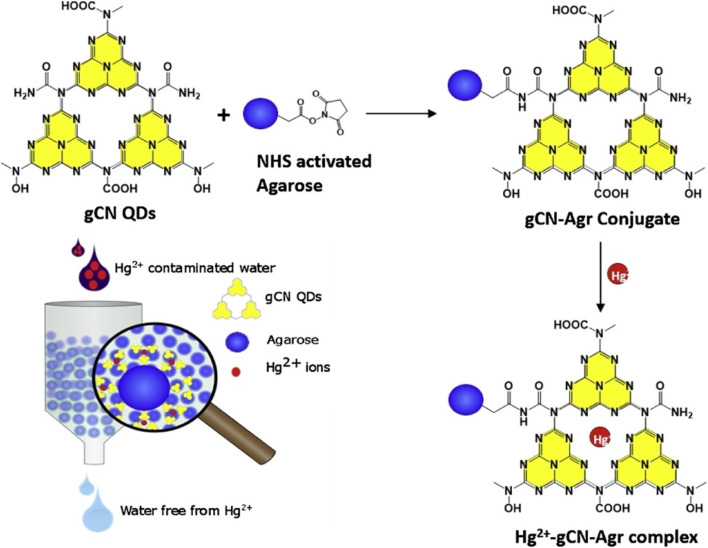
Schematic of the synthesis of gCNQDs and Hg^2+^ sensing. This figure has been adapted from ref. 108 with permission from Elsevier, copyright 2019.

Chadha *et al.* reported the synthesis of water-stable gCN QDs (Scheme 21) *via* a microwave-assisted solvothermal route and characterized them through fluorescence, FT-IR, XRD, HR-TEM, and Raman spectroscopy. The QDs exhibited excitation-dependent fluorescence that was significantly quenched in the presence of Hg^2+^; this was corroborated by DFT calculations, which revealed that the embedment of mercury atoms onto the synthesized gCN surface led to structural distortion and band gap reduction. Notably, the strong Hg^2+^ binding affinity of graphitic carbon nitride quantum dots (g-CN QDs) was further exploited in the development of a bioinspired micro-cartridge system through covalent conjugation with agarose microbeads. The resulting micro-cartridge demonstrated effective removal of mercury from contaminated water, exhibiting a binding capacity of 24.63 mg HgCl_2_ per 10 mg of agarose-g-CN conjugate. The cartridge-based device loaded with g-CN QDs enabled efficient capture of Hg^2+^ ions from aqueous samples, highlighting its potential applicability in environmental remediation and related biomedical contexts. In addition, the g-CN QDs were shown to serve as highly fluorescent and biocompatible probes for Hg^2+^ quantification over a broad concentration range of 50 ppb to 50 ppm. The metal-binding capability of g-CN, corroborated by DFT studies, was further leveraged to fabricate agarose-based micro-columns, achieving mercury removal efficiencies of up to 98% in spiked real water samples. Importantly, the reported synthetic route allows straightforward scale-up, enabling the large-scale preparation of such micro-columns for the efficient capture of mercury ions from contaminated water sources and offering a promising strategy for improving water safety.^108^

In another report, a photoelectrochemical (PEC) sensor was developed using a g-C_3_N_4_/CdS QD composite immobilized on an FTO electrode g-C_3_N_4_@CdS/FTO for sensing of Hg^2+^. The system exhibited a linear range of 20–550 nM, with a LOD of 12 nM. The detection principle relied on “signal shutdown”, wherein Hg^2+^ competes with Cd^2+^ to form HgS. Given the much smaller solubility product of HgS (*K*_sp_ = 4 × 10^−53^) compared to CdS (8 × 10^−27^), exciton generation and electron transport were inhibited, leading to photocurrent quenching. Practical applicability was demonstrated by recoveries of 96–103% in tap and lake water samples, with low RSD, confirming the reliability of the sensor. The proposed “signal shutdown” strategy demonstrated reliable analytical performance and broadened the applicability of photoelectrochemical (PEC) sensing to real water matrices, including tap and lake water. The measured Hg^2+^ concentrations in the tested samples were in good agreement with the spiked values, yielding recovery rates in the range of 96–103% and low relative standard deviation (RSD) values. These results indicate that the developed PEC sensor possesses promising potential for the determination of Hg^2+^ in aqueous environments.^109^ Similarly, a facile “bottle-around-ship” strategy has been employed to construct a ratiometric fluorescent probe by co-encapsulating red-emissive CdTe quantum dots and blue-emissive graphitic carbon nitride quantum dots (g-CNQDs) within a zeolitic imidazolate framework (ZIF-8). Upon excitation at 360 nm, the resulting ZIF-8@g-CNQD/CdTe composite exhibited dual emission bands centered at 450 and 633 nm. In the presence of Hg^2+^ ions, the red emission of CdTe QDs was selectively quenched, while the blue emission from g-CNQDs remained largely unaffected and served as an internal reference, leading to a visible color change from pink to blue. The ratiometric fluorescence signal (*F*_633_/*F*_450_) decreased linearly with increasing Hg^2+^ concentration over the range of 0.2–3.5 µmol L^−1^, with a LOD of ∼46 nmol L^−1^. Furthermore, the probe demonstrated reliable performance for Hg^2+^ determination in real sample matrices.^110^

Similarly, Li *et al.* developed a label-free, self-enhanced electrochemiluminescence (ECL) sensing platform for Hg(ii) detection. The system was based on a novel luminophore, prepared by combining Ru(bpy)_3_^2+^ with carbon nitride quantum dots (CNQDs) *via* electrostatic interaction, thereby forming a self-enhanced ECL reagent. Unlike conventional ECL systems that rely on intermolecular reaction between the emitter and its co-reactant, the self-enhanced ECL system demonstrated a reduced electron-transfer distance and improved luminescence efficiency, as electrons were transferred from the CNQDs to oxidized Ru(bpy)_3_^2+^ through an intramolecular pathway. Encapsulation within SiO_2_ NPs to generate a Ru-QDs@SiO_2_ luminophore further enhanced stability. Exploiting the differential affinity of Ru-QDs@SiO_2_ nanoparticles toward single-stranded DNA (ssDNA) and Hg^2+^-induced double-stranded DNA (dsDNA), a fluorescence biosensor was developed that exhibited high sensitivity for Hg^2+^ detection. The system achieved a low LOD of 33 pM over a wide linear concentration range of 0.1 nM–10 µmol L^−1^. Its practical applicability was further confirmed through the successful analysis of spiked water samples using the fabricated biosensor.^111^ In a related study, Pattnayak *et al.* reported silver nanoparticle-embedded sulfur-doped graphitic carbon nitride quantum dots (Ag–S–gCN QDs) as a fluorescent probe for Hg^2+^ sensing. The as-prepared quantum dots, with an average particle size of approximately 3.7 nm, exhibited intense blue fluorescence with a relative quantum yield of 36.5%, and exhibited strong blue emission along with notable photostability. Under optimized conditions (pH 5), the Ag–S–gCN QDs nanosensor achieved rapid sensing of Hg^2+^ ions (3 minutes) with a LOD of 0.13 µM and LOQ of 0.43 µM across a linear range of 0.1–0.6 µM. Time-resolved fluorescence studies (lifetime 7.79 ns) indicated a static quenching mechanism involving electron transfer from metallic Ag to Hg^2+^, consistent with redox-mediated interactions. Practical relevance was demonstrated by recoveries exceeding 85% in real water samples, with RSD ≤ 5%. The proposed nanosensor is expected to open new avenues for the convenient, efficient, sensitive, and selective detection of potentially hazardous Hg^2+^ ions. Furthermore, its practical applicability was validated through Hg^2+^ determination in tap and lake water samples, achieving satisfactory recovery values and high analytical precision. While the incorporation of Ag into sulfur-doped gCN QDs enhances electron-transfer pathways and imparts rapid sensing capability, the detection limit is modest compared to more advanced quantum-dot-based systems that achieve nanomolar or even picomolar sensitivity. Additionally, the narrow linear detection range and pH-dependence (optimal at pH 5) may limit versatility in diverse environmental matrices.^76^

**Scheme 22:**
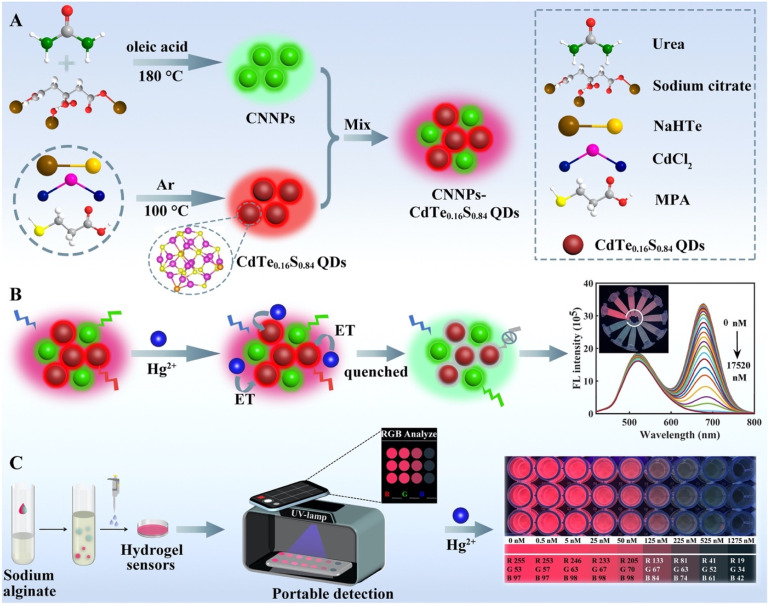
Schematic of (A) the preparation of CNNPs-CdTe_0.16_S_0.84_ QDs, (B) the ET quenching mechanism of CNNPs-CdTe_0.16_S_0.84_ QDs for Hg^2+^, and (C) a hydrogel sensor with smartphone detection of Hg^2+^. This figure has been adapted from ref. 8 with permission from Elsevier, copyright 2024.

A ratiometric fluorescence sensing platform was developed using CN nanoparticles (CNNPs) as a green, green-emitting self-calibration signal and CdTe_0.16_S_0.84_QDs as a red-emitting response signal for rapid Hg^2+^ detection (Scheme 22A). This new fluorescence sensor exhibited a broad linear range (0.05–25 nM) and a LOD of 0.009 nM. The sensing mechanism relied on electron transfer (ET) and electrostatic interactions between Hg^2+^and CdTe_0.16_S_0.84_ QDs, which disrupted radiative recombination, resulting in fluorescence quenching. The foremost advantages of this ratiometric fluorescence sensing include: (1) a straightforward probe synthesis, accompanied by a high fluorescence quantum yield, excellent optical properties, and good chemical stability; (2) wide detection and ultra-low LOD; and (3) integration of the ratiometric probe with a hydrogel and smartphone-based imaging that enabled portable and intelligent on-site monitoring of Hg^2+^ in environmental water samples, validated with good recovery in lake water. However, the reliance on CdTe-based QDs raises concerns regarding toxicity and environmental sustainability, which could hinder large-scale deployment.^8^

Chen *et al.* reported a cost-effective graphitic carbon nitride quantum dot (g-CNQD)-based fluorescent platform for the rapid detection of Hg^2+^ and H_2_O_2_. The sensing mechanism is governed by fluorescence quenching arising from interactions between the g-CNQDs and the target analytes. In the presence of KI, the g-CNQDs exhibited concentration-dependent fluorescence quenching toward H_2_O_2_ over a wide linear range of 1–1000 µmol L^−1^, with a LOD of 0.23 µmol L^−1^. In addition, the system demonstrated high sensitivity toward Hg^2+^, showing a linear detection range of 0–0.1 µmol L^−1^ and a LOD of 0.038 µmol L^−1^. The ability of g-CNQDs to enable dual-analyte detection highlights their practical applicability and underscores their potential as versatile platforms for the development of bifunctional or multifunctional sensing systems.^112^ Thara *et al.* synthesized carbon nitride dots (CNDs) *via* a one-step method using ascorbic acid and urea as precursors. HRTEM analysis revealed quasi-spherical nanoparticles with an average size of ∼3.11 nm. FTIR spectroscopy showed broad absorption bands in the range of 3400–3000 cm^−1^, attributed to N–H and O–H stretching vibrations, while characteristic bands at 1570 and 1410 cm^−1^ corresponded to C–N stretching modes. In addition, the presence of a cyano functional group was confirmed by an absorption band at 2193 cm^−1^. Electrochemical investigations demonstrated that the CNDs exhibited high selectivity and sensitivity toward Hg^2+^ ions and the antibiotic tetracycline (TC) in both laboratory and real water samples, with LODs of 0.055 µmol L^−1^ and 0.18 µmol L^−1^, respectively. Cytotoxicity studies further revealed pronounced toxicity of the CNDs toward cancer cells relative to fibroblast cells, indicating dual-mode anticancer activity arising from both chemotherapeutic and photodynamic effects. These findings suggest that the one-step-synthesized carbon nitride dots can serve as efficient electrochemical sensors for Hg^2+^ and TC detection, while also showing promise as anticancer nanomedicines. The applicability of the fabricated electrode was validated using differential pulse voltammetry (DPV), achieving reliable detection of Hg^2+^ and TC in spiked real water samples with relative standard deviation values ranging from 0.003% to 0.40%.^113^

A binary composite of NH_2_-UiO-66 and g-CNQDs was synthesized to leverage the porosity and stability of the MOF framework along with the excellent electronic properties of g-CNQDs. Incorporation of g-CNQs onto the porous MOF surface enhances electronic stability by suppressing the recombination of photogenerated electron–hole pairs, thereby prolonging the fluorescence lifetime of photoinduced carriers and boosting fluorescence emission. The enhanced fluorescence of the synthesized binary MOF composite arises from the synergistic effect of electron-rich surface groups from the g-CNQs and the –NH_2_-BDC linker, which together promote efficient electronic interactions and emission. The resulting NH_2_-UiO-66/g-CNQDs composite exhibited a significantly low LOD of 2.4 nmol L^−1^ (∼0.0006 mg L^−1^), which is below the WHO permissible limit, and the Stern–Volmer constant *K*_sv_ was ∼2.3 × 10^7^ L mol^−1^. The mechanistic studies indicated dynamic fluorescence quenching *via* a photoinduced electron transfer (PET) process, supported by reduced lifetimes (25.75 → 5.44 ns) upon Hg^2+^ addition. PXRD and FTIR analyses confirmed the absence of structural changes or direct bonding interactions, suggesting that Hg^2+^ diffuses freely into MOF channels and induces fast response behavior. The practical applicability of the sensor was further evaluated through real water sample analysis, confirming that the synthesized binary composite functions as an efficient platform for the detection of Hg^2+^ ions in real water matrices.^77^

Guo *et al.* developed a graphitic carbon nitride nanosheet-based nanocomposite incorporating graphitic carbon nitride quantum dots (CNQDs/CNNNs) through a one-step pyrolysis strategy. The as-prepared material exhibited good thermal stability as well as resistance to photobleaching and high salt concentrations. Mechanistic insights obtained from XPS, UV-Vis diffuse reflectance spectroscopy, and DFT calculations suggested that Hg^2+^ ions interact with the CNQDs/CNNNs architecture, inducing structural variations that alter the bandgap and result in fluorescence quenching. The sensing system showed a linear response toward Hg^2+^ in the concentration range of 0.025–4.0 µmol L^−1^, with a LOD of 7.82 nmol L^−1^. These features highlight the potential of CNQDs/CNNNs-based platforms for the quantitative determination of Hg^2+^ in environmental water samples.^114^

### 3.5 g-C_3_N_4_@carbon based sensors for mercury detection

**Fig. 18:**
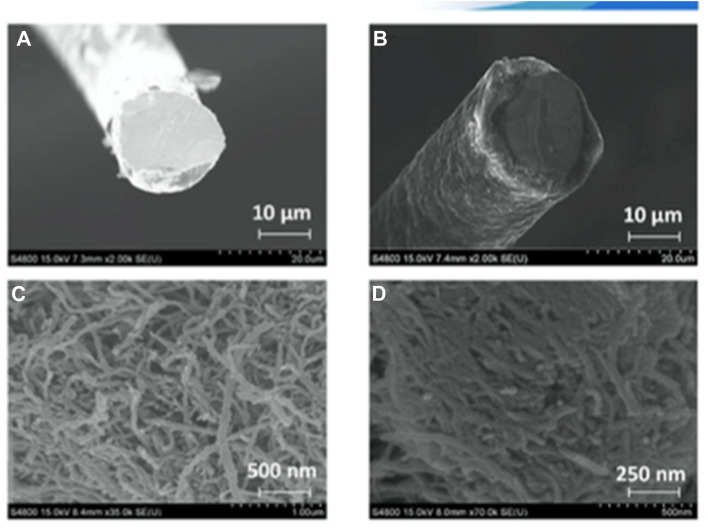
SEM image of (A) bare carbon fiber, (B) carbon fiber disk microelectrode, and (C) MWCNT formed framework on a carbon fiber disk microelectrode. (D) P-CN_T60 assembled on the MWCNT formed framework on the carbon fiber disk microelectrode. This figure has been adapted from ref. 115 with permission from Elsevier, copyright 2017.

The integration of g-C_3_N_4_ with carbon-based nanostructures has emerged as a promising strategy to enhance mercury ion (Hg^2+^) sensing. While pristine g-C_3_N_4_ is associated with limited surface area and poor charge transport, coupling with conductive carbon materials, such as carbon nanotubes (CNTs), graphene oxide (GO), reduced graphene oxide (rGO), and carbon dots (CDs), significantly improves sensitivity, selectivity, and stability. These hybrids benefit from (i) the high electrical conductivity of carbon materials, (ii) abundant surface functional groups for Hg^2+^ coordination, and (iii) synergistic fluorescence or electrochemical responses. For instance, H. Lv *et al.* developed oxygen-functionalized carbon nitride nanosheets assembled with multi-walled carbon nanotubes on a carbon fiber disk microelectrode, which exhibited remarkable sensitivity towards Hg^2+^ along with Cu^2+^ and Pb^2+^. The morphology and electrochemical properties of CNT60/MWCNT/CFE were characterized by SEM, CV and ASDPV. The SEM images (Fig. 18) confirmed the smooth surface of the bare carbon fiber, which, after electrochemical deposition and modification with MWC-NTs, CN T60, showed a net-like porous framework. TEM further verified the uniform assembly. The fabricated electrode demonstrated ultra-high sensitivity for simultaneous detection of Cu^2+^, Pb^2+^ and Hg^2+^, with LOD values of 1.0 × 10^−13^ mol L^−1^ for Cu^2+^, 1.8 × 10^−11^ mol L^−1^ for Pb^2+^ and 8.0 × 10^−12^ mol L^−1^ for Hg^2+^, respectively. The linear ranges extended up to ∼8.5 × 10^−6^ mol L^−1^ for all three metal ions. More crucially, P-CN T60/MWCNT/CFE can be renewed for additional examination at least five times, and its cost-effectiveness (∼$5 per electrode) makes it highly suitable for practical applications, including mechanistic studies of metal uptake in biological systems, such as rice roots, at trace levels. However, while simultaneous detection of multiple metal ions is advantageous, possible interference between analytes under real environmental conditions remains a challenge that requires further validation.^115^

**Scheme 23:**
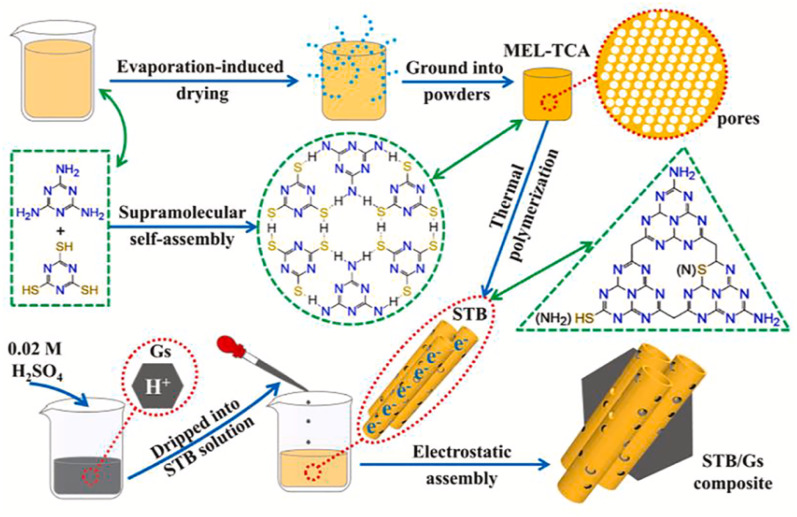
Schematic of the preparation of STB/Gs composite. This figure has been adapted from ref. 117 with permission from Elsevier, copyright 2021.

**Fig. 19:**
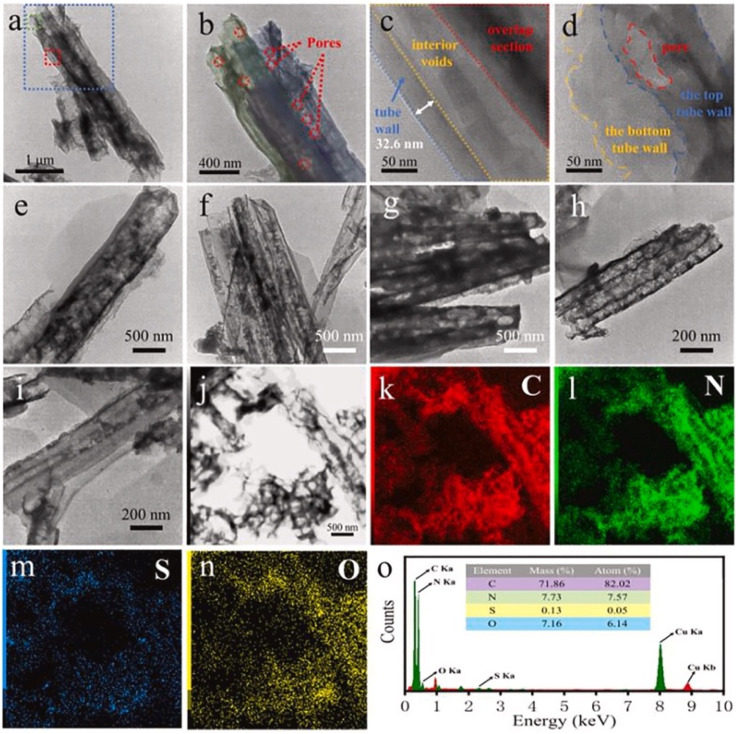
TEM images of (a–d) STB and (e–i) STB/Gs-2, and (j–n) STEM image and EDS elemental maps of STB/Gs-2 for C (k), N (l), S (m), O (n) elements. (o) EDS spectrum of STB/Gs-2. This figure has been adapted from ref. 117 with permission from Elsevier, copyright 2021.

Similarly, in a study, a notable advancement was reported when a 3D nanocomposite comprising porous g-C_3_N_4_ nanosheets (p-g-C_3_N_4_-NSs) and oxidized multiwalled carbon nanotubes (O-MWCNTs) was obtained *via* a one-step simultaneous chemical oxidation process. This strategy not only generated acidic functional groups on both components but also enabled *in situ* incorporation of O-MWCNTs into the p-g-C_3_N_4_ framework, thereby enhancing surface area and electron-transport pathways. The nanocomposite was used to modify a screen-printed electrode (SPE) for electrochemical studies. The hybrid nanocomposite revealed remarkable selectivity for simultaneous detection of Cd(ii), Hg(ii), Pb(ii) and Zn(ii), achieving detection limits as low as 8–60 ngL^−1^ through stripping analysis. Notably, the method was utilized for the concurrent detection of these ions in diverse (spiked) food samples. The results demonstrated the good accuracy and reproducibility of the method. However, despite the impressive sensitivity and multi-ion detection capability, the study does not fully address challenges such as electrode fouling in complex matrices, long-term sensor stability, or scalability of the synthesis.^116^ A recent study explored the synergistic interaction of sulfur-doped C_3_N_4_ tube bundles (STB) with hierarchical pores and graphene nanosheets (Gs) to achieve ultra-trace HMIs detection (Scheme 23). The hierarchical pores expedited the rapid diffusion and adsorption of sulfur active sites (Fig. 19), while graphene nanosheets provided rapid electron transport, thereby accelerating the redox deposition and stripping of Cd^2+^, Pb^2+^ and Hg^2+^. Under optimum experimental settings, the STB/Gs-2 composite achieved remarkable LODs of 1.17, 0.38 and 0.61 nM for concurrent detection and 2.30, 0.78 and 1.15 nM for distinct detection. The STB/Gs-2 electrodes exhibited an expanded linear range, along with good stability, anti-interference resistance, and repeatability. The practical applicability was validated *via* simultaneous detection of Cd^2+^, Pb^2+^ and Hg^2+^ in real water samples. Collectively, the results indicate that the STB/Gs-2 composite represents a promising C_3_N_4_-based electrochemical platform for environmental monitoring. Despite these advantages, the practical deployment of such hybrid materials may be limited by the complexity of tube bundle synthesis, possible electrode fouling in real matrices, and the scalability of STB/Gs composites.^117^

An innovative approach employed phosphorus doping to convert hollow-tube polymeric carbon nitride into oligomeric needle-like nanostructures enriched with surface amino and hydroxyl groups. This structural transformation significantly enhanced metal–ligand interactions, leading to highly selective fluorescence quenching of Hg^2+^ with a significant LOD of 1.14 nM. The study highlights the potential of phosphorus doping for tailoring the surface chemistry and improving detection sensitivity. However, the work did not thoroughly examine the sensor's performance in complex environmental samples or its long-term stability, which are crucial for practical applications. Thus, while promising, additional validation is required to translate this oligomer-based sensor into practical mercury-monitoring platforms.^118^

### 3.6 g-C_3_N_4_@polymers and other materials for mercury sensing

**Fig. 20:**
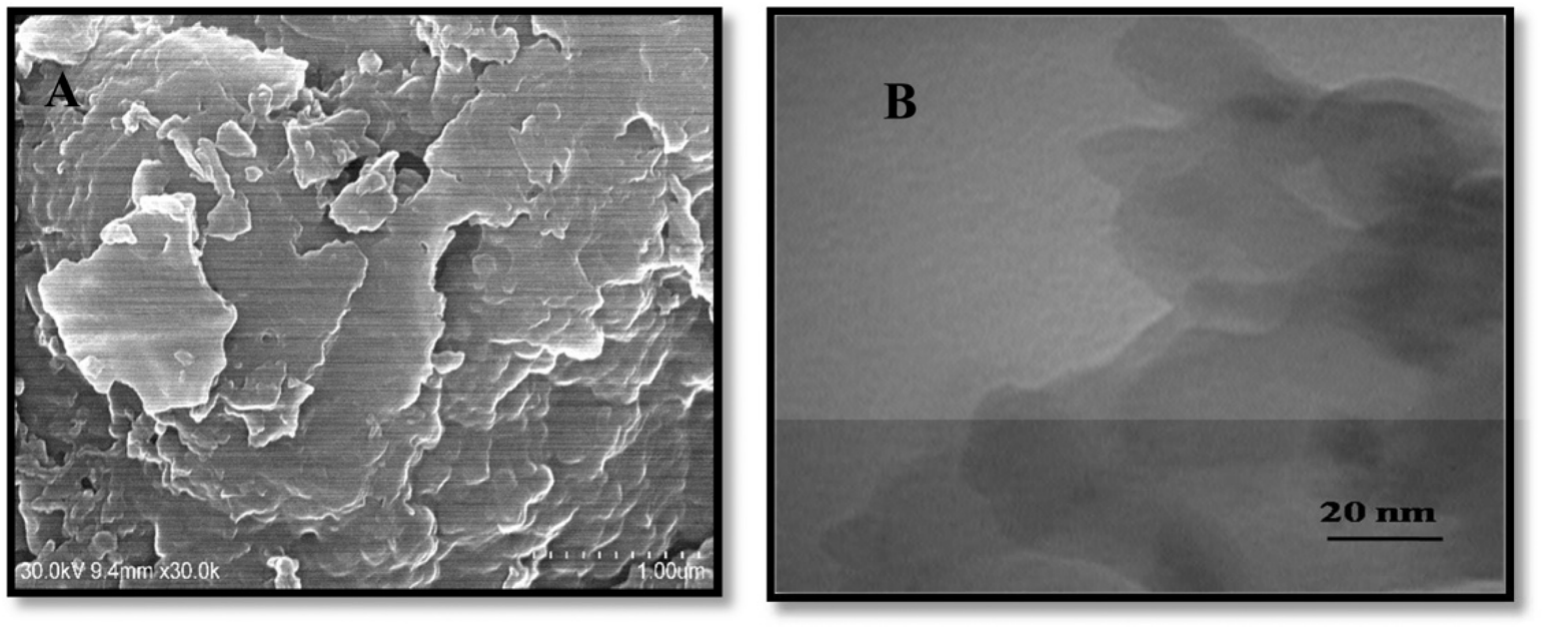
(A) SEM image of g-C_3_N_4_ and (B) TEM image of g-C_3_N_4_. This figure has been adapted from ref. 119 with permission from Elsevier, copyright 2016.

The incorporation of g-C_3_N_4_ with polymers and other well-designed materials offers a further class of rational materials for mercury sensing that enable tailored physicochemical properties, increased active sites, and improved interaction between Hg^2+^ ions and the sensing interface. For instance, a carbon paste electrode, prepared *via* the casting method and modified with g-C_3_N_4_/chitosan composite, has been reported for Hg^2+^ detection. Microscopic analysis confirmed the regular sheet-like morphology of the hybrid material (Fig. 20), while FT-IR analysis validates the structural features of the g-C_3_N_4_, including heptazine arrangement, and the terminal NH_2_ or NH groups. Electrochemical sensing studies revealed two linear response ranges over 1.0 × 10^−6^ to 8.0 × 10^−5^ mol L^−1^ and 1.0 × 10^−7^ to 5.0 × 10^−6^ mol L^−1^ with a LOD of 1.0 × 10^−8^ mol L^−1^. Notably, the platform offered satisfactory selectivity and accuracy for Hg(ii) sensing, with recoveries of 98.65% and 102.03%. The reliability of the g-C_3_N_4_/chitosan composite was examined for two real water samples. The incorporation of chitosan appears to enhance adsorption and electron transfer, suggesting that modification of g-C_3_N_4_ with a biopolymer is an effective strategy to improve selectivity and sensitivity compared to pristine g-C_3_N_4_-based electrodes.^119^

**Fig. 21:**
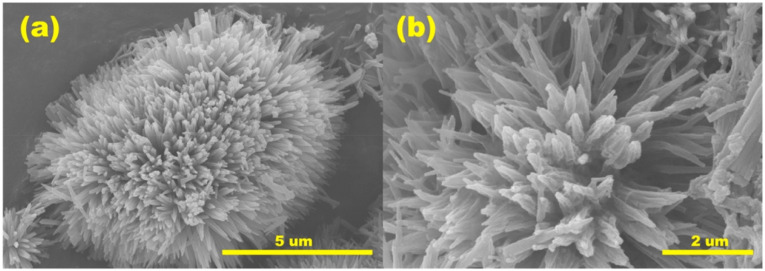
(a) and (b) SEM images of P-DPCN. This figure has been adapted from ref. 121 with permission from Elsevier, copyright 2021.

In a report, NPs of an ion-imprinted polymer (IIPs) have also been employed in combination with g-C_3_N_4_ to construct highly selective electrochemical sensors for Hg(ii), consuming itaconic acid as a functional monomer *via* precipitation polymerization. The existence of g-C_3_N_4_ considerably improved the adsorption and electron-transfer properties, while the IIPs provided specific binding sites for Hg(ii), resulting in improved selectivity compared to the unmodified electrodes. Using square wave anodic stripping voltammetry (SWASV), the sensor achieved an inclusive linear range of 0.06–25.0 nM and an LOD of 18 pM, outperforming many conventional g-C_3_N_4_-based structures. Validation in real water samples confirmed its reliability, highlighting that the synergistic combination of IIPs and g-C_3_N_4_ represents a promising strategy for ultrasensitive and selective mercury detection.^120^ The fluorescence quenching effect of polymeric carbon nitride (PCN) based materials was efficiently exploited by Zhao *et al.* for the rapid detection of Hg^2+^ ions. The morphological analysis (SEM, Fig. 21, and TEM) revealed that P-DPCN consists of nanorods (diameter ∼50 nm and respective microns in length), which aggregated into a sea-urchin structure upon freeze-drying. Importantly, P-DPCN exhibited strong fluorescence emission, high quantum yield, and a pronounced quenching response toward Hg^2+^. The sensor demonstrated a linear response in the range of 2–50 nmol L^−1^ with a LOD of 0.74 nmol L^−1^, surpassing many previously reported methods. While these results highlight the potential of P-DPCN as a favorable sensing material for mercury detection in actual samples, the long-term stability of the nanorod structures in aqueous environments and possible interference from other competing ions require further exploration for practical deployment.^121^

**Scheme 24:**
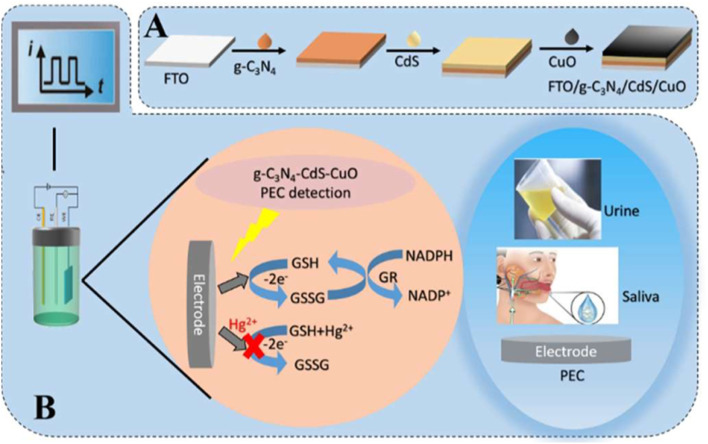
Schematic of (A) the electrode preparation process, and (B) the redox cycle on the photoanode of a self-powered device and tests with an actual sample. This figure has been adapted from ref. 123 with permission from MDPI, copyright 2022.

In a comparable approach, a P-doped carbon nitride oligomer-based fluorescent probe was reported for selective sensing of mercury ion, exhibiting a quantum yield of 16.6% and an ultralow LOD of 0.35 nmol L^−1^ in the range of 1–100 nmol L^−1^. The applicability of the probe was validated *via* mercury detection in actual shrimp samples, and the results validated the satisfactory sensitivity and accuracy. This work highlights that the P-doped g-C_3_N_4_ derived oligomer provides new prospects for developing sustainable and cost-effective fluorescent sensing platforms for mercury sensing.^122^ Similarly, in another study, a self-powered photoelectrochemical (spPEC) sensing platform was developed for Hg^2+^ sensing using a g-C_3_N_4_–CdS–CuO co-sensitized photoelectrode coupled with a visible-light-induced redox cycle intensification strategy. The sequential assembly of single-layer g-C_3_N_4_, CdS, and CuO onto the electrode surface resulted in a pronounced enhancement in PEC activity. Under optimal conditions, the photocurrent achieved an LOD of 0.84 pM for mercury ions over a linear concentration range of 5 pM–100 nM. Furthermore, the sensor exhibited reliable performance for mercury analysis in artificial saliva and human urine. More appreciably, the approach can be extended to detect other heavy metal ions based on the HSAB principle, offering a versatile platform for applications in food, clinical toxicology, and the environment (Scheme 24).^123^

**Scheme 25:**
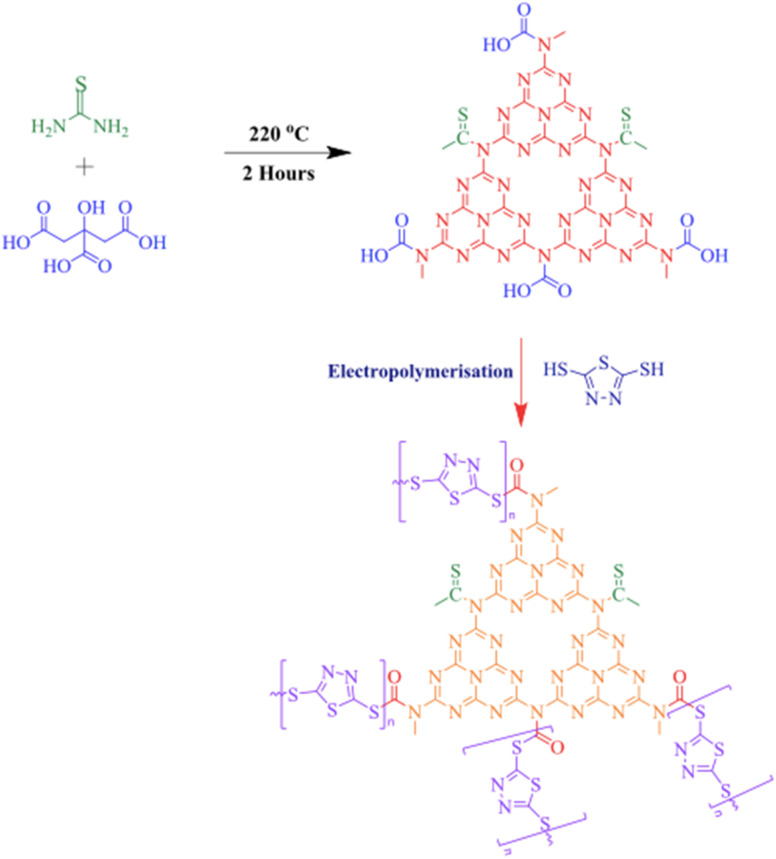
Schematic of the synthesis of S, O-GCN@PTD. This figure has been adapted from ref. 25 with permission from Elsevier, copyright 2023.

**Fig. 22:**
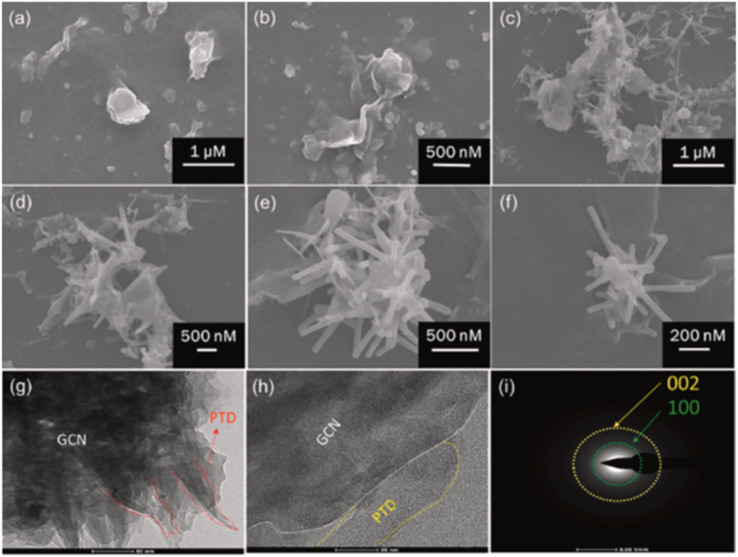
FESEM images of (a–f) S, O-GCN and (d–f) S, O-GCN@PTD, (g and h) HRTEM images of S, O-GCN@PTD, and (i) SEAD pattern. This figure has been adapted from ref. 25 with permission from Elsevier, copyright 2023.

In a study, screen-printed carbon electrodes (SPCE) were tailored with S- and O-incorporated g-C_3_N_4_ (S, O-GCN) linked poly(1,3,4-thiadiazole-2,5-dithiol) film (PTD) *via* a thioester linkage (Scheme 25). XRD analysis confirmed the crystalline nature of S, O-GCN, showing diffraction peaks at 13.07° and 27.3°, corresponding to the (1 0 0) and (0 0 2) planes of the GCN stacking. Interestingly, the absence of polymer-related peaks in S, O-GCN@PTD suggested the successful incorporation of the polymer film with high purity. FE-SEM images (Fig. 22) further revealed that O- and S-functionalization induced crinkled flake morphologies, while the PTD coating promoted nanotube- and rod-like structures on the flakes, thereby enhancing the surface area and increasing the number of active sites for Hg^2+^ binding. Electrochemically, the synergistic presence of sulfur and oxygen atoms provided a strong affinity towards Hg^2+^, which was studied by DPASV. The S, O-GCN@PTD-SPCE achieved electrochemical signals from Hg^2+^ ions over the concentration range of 0.05–390 nM, with a LOD of 13 pM. The electrode was further validated in different environmental samples, including tap water, river water and food samples (fish and crab). The results were confirmed with ICP-OES analysis, which indicated values that were practically the same. While the study highlights the remarkable sensitivity and real-sample applicability of S, O-GCN@PTD-SPCE, critical challenges remain regarding electrode reproducibility, long-term stability under complex matrices, and potential interference from coexisting heavy metals. Addressing these aspects is crucial for translating such high-performance nanostructured electrodes from laboratory demonstrations to field-deployable mercury sensors.^25^

**Fig. 23:**
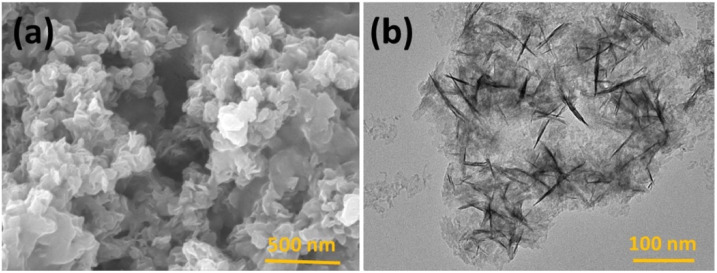
(a) SEM and (b) TEM images of polymeric CN nanosheets. This figure has been adapted from ref. 10 with permission from RSC, copyright 2024.

In another report, polymeric g-C_3_N_4_ nanosheets have also been explored as solid-phase extraction (SPE) sorbents for the enrichment of trace Hg(ii) ions. SEM and TEM analyses (Fig. 23) confirmed the layered and wrinkled morphology of exfoliated nanosheets, while BET measurements revealed a relatively high surface area (158 m^2^ g^−1^) and good porosity (0.864 cm^3^ g^−1^). The nanosheets displayed a negative zeta potential (−20 mV), favoring strong electrostatic interactions with positively charged Hg(ii) species, which were further stabilized through coordination with nitrogen atoms in the polytriazine and heptazine frameworks. Analytically, the material demonstrated a preconcentration limit of 0.33 mg L^−1^ and a high enrichment factor of 200, enabling detection of trace Hg(ii) in real water samples with good precision (RSD < 5%). However, the reported LOD of 0.6 mg L^−1^ is relatively high compared to state-of-the-art electrochemical or fluorescence-based g-C_3_N_4_ sensors, which often achieve detection in the nanomolar or even picomolar range. While the SPE-based approach is attractive for preconcentration and matrix clean-up, its relatively modest sensitivity limits its standalone applicability for trace-level mercury monitoring. Future directions could focus on coupling g-C_3_N_4_ SPE sorbents with more sensitive detection platforms (*e.g.*, ICP-MS and electrochemical stripping analyses) to overcome this drawback and expand their practical utility.^10^

**Fig. 24:**
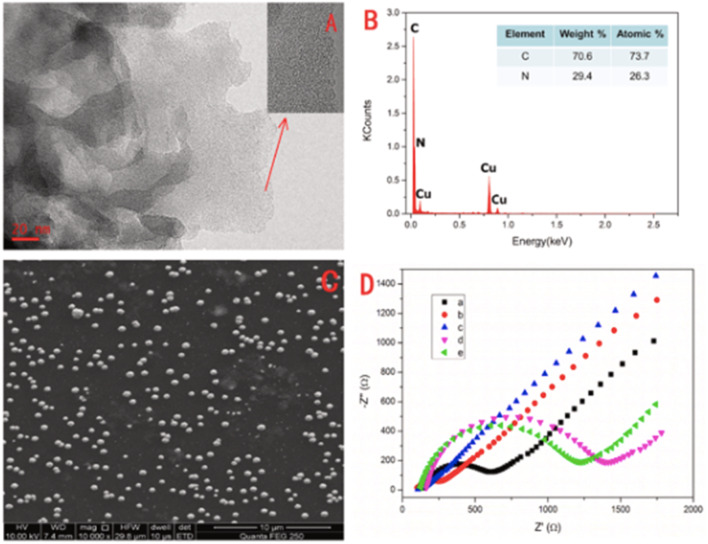
(A) and (B) TEM image and XRD of C-g-C_3_N_4_ (C) SEM image of C-g-C_3_N_4_/Eau, and (D) EIS of bare GCE. This figure has been adapted from ref. 124 with permission from Elsevier, copyright 2024.

**Scheme 26:**
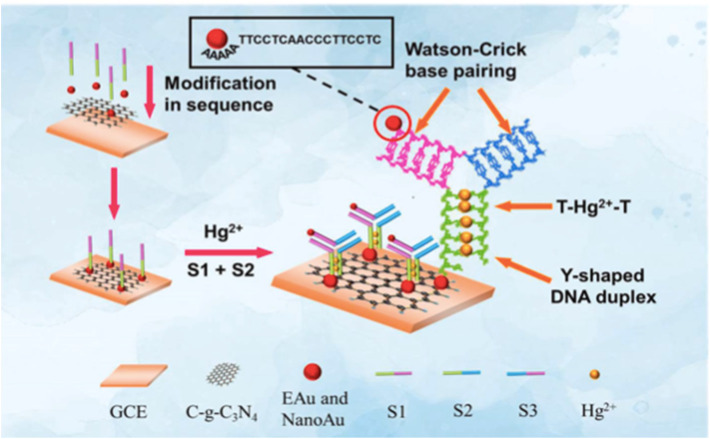
Schematic of the DNA sensor's sensing response for Hg^2+^ ions. This figure has been adapted from ref. 124 with permission from Elsevier, copyright 2024.

A DNA electrochemical sensor employing Y-shaped DNA probes was developed by Y. Zhu *et al.* for Hg^2+^ detection. Carbon self-doping graphite-like carbon nitride (C-g-C_3_N_4_) and electrodeposited gold nanoparticles (EAu) were integrated onto a glassy carbon electrode to enhance conductivity and provide abundant binding sites for DNA immobilization. TEM and EDX (Fig. 24) analyses confirmed the successful carbon doping in g-C_3_N_4_, while SEM revealed the uniform decoration of large gold NPs (∼500 nm) on the nanosheets. Electrochemical resistivity further demonstrated that the C-g-C_3_N_4_/EAu interface greatly reduced charge-transfer resistance, highlighting the strong synergistic effect of these modifiers in facilitating electron transport. The sensing mechanism relied on the formation of a rigid Y-shaped DNA duplex that specifically captured Hg^2+^ through thymine–Hg^2+^–thymine interactions, thereby amplifying the electrochemical response (Scheme 26). The sensor exhibited an impressive detection range (5.0 nM–5.0 µM) with a remarkably low LOD of 8.5 pM. Real-sample analyses, including tap, river, and medical wastewater, further confirmed its practical applicability, with high recovery and accuracy. This strategy demonstrates the advantage of combining self-doped g-C_3_N_4_ with noble-metal nanoparticles to overcome conductivity limitations and support biomolecular assemblies. However, some challenges remain: the relatively large size of the electrodeposited gold particles may reduce the surface area efficiency compared to its nanoscale counterparts, and the reliance on DNA probes increases fabrication cost and potential storage instability.^124^

**Fig. 25:**
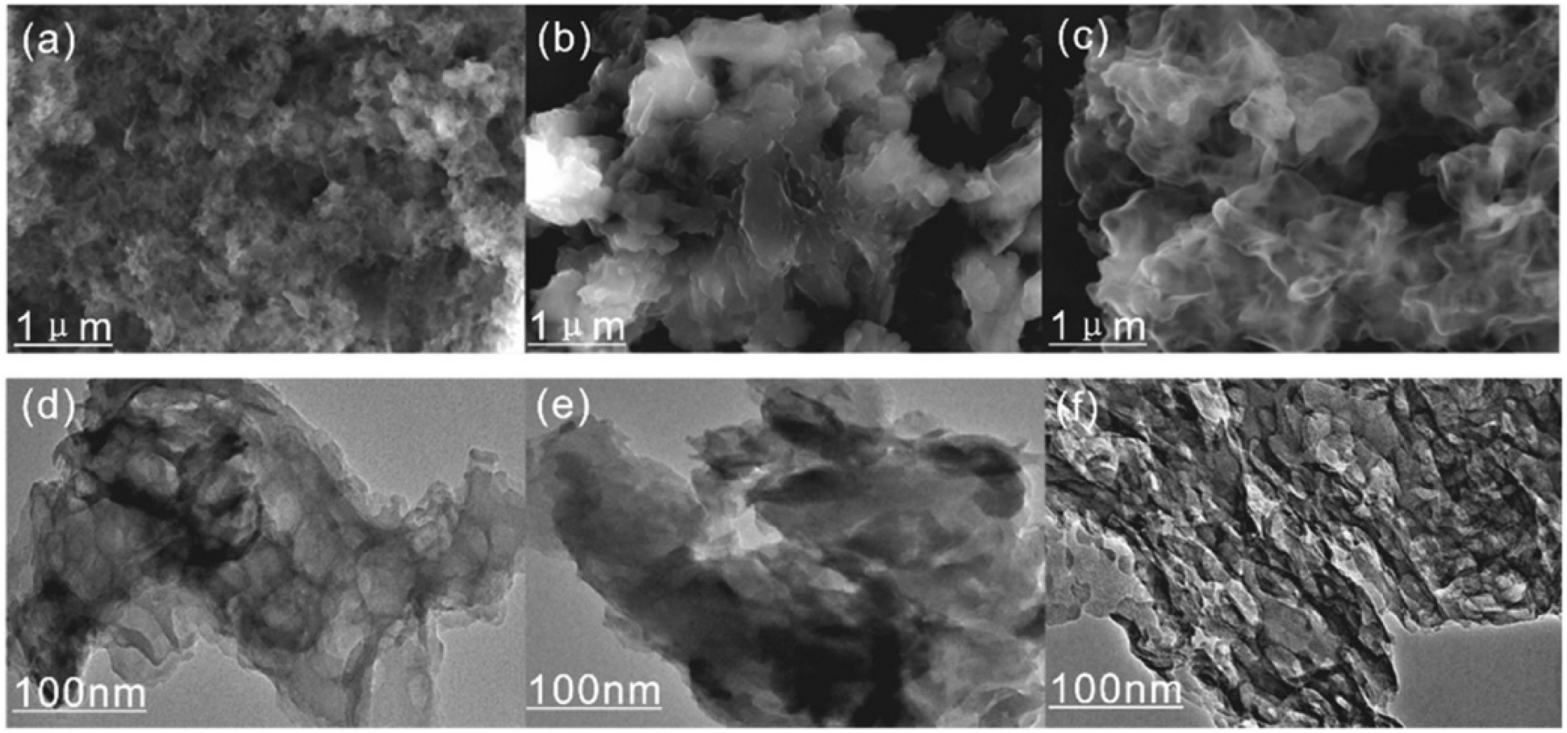
SEM images of (a) U-gCN, (b) T-gCN, and (c) UT-gCN (3 : 1), and TEM images of (d) U-gCN, (e) T-gCN, and (f) UT-gCN (3 : 1). This figure has been adapted from ref. 125 with permission from Elsevier, copyright 2021.

An electrochemical sensor was developed by X. Chen *et al.* for the detection of methylmercury based on Au electrodes modified with sulfur-doped g-C_3_N_4_ (S-g-C_3_N_4_) heterojunctions. Structural characterization (Fig. 25) confirmed well-defined porous morphologies, layered microstructures and uniform elemental distribution of the S and O dopants, which collectively enhanced the charge transfer and suppressed electron–hole recombination. The sensor demonstrated high analytical performance for methylmercury, with a linear response of 0–25 ppb CH_3_Hg^+^, a sensitivity of 0.52 µA ppb^−1^, and a 0.175 ppb detection limit. Importantly, the electrochemical sensor is robust and does not exhibit interference with Cu^2+^, Cd^2+^, Pb^2+^, Bi^3+^, or As^3+^, making it promising for real-world applications. Additionally, sensors could be replenished by protonation with HCl, offering reusability. From a broader perspective, this sensor highlights the potential of heteroatom-doped g-C_3_N_4_ in electrochemical sensing of methylmercury and more toxic organic species, rather than targeting only Hg^2+^, which is the focus of most g-C_3_N_4_-based sensors. However, despite the high sensitivity and low LOD for methylmercury, interferences from organic matter or competing sulfur species may influence the performance.^125^ To regulate the interferences from organic matter and competing sulfur species, some practices can be done; (I) doping with transitional metals (*e.g.*, Zn, Fe), which generates active sites that resist salt precipitation, (II) surface modification and functionalization, which introduces oxygen-containing groups (carboxyl, hydroxyl) through controlled oxidation, enhances hydrophilicity, and provides selectivity against organic foulants.^71,76^

Sulfur-doped g-C_3_N_4_ was synthesized *via* one-pot hydrothermal mixing of urea and thiourea and utilized as a potentiometric sensor *via* fabrication on a carbon paste electrode for Hg^2+^ detection. Electrochemical sensing demonstrated that the electrode impregnated with S-g-C_3_N_4_, presented remarkable sensitivity over a concentration range of 1 × 10^−7^ to 1 × 10^−2^ mol L^−1^, with an LOD of 6.5 × 10^−8^ mol L^−1^ for mercury ions. Notably, the electrode offered long-term stability (>5 months) with a fast response time of 50 s. The most imperative observation was that no substantial effect was observed for the potential response of the S-g-C_3_N_4_-modified CPE to interfering ions (Cu^2+^, Pb^2+^, Ni^2+^ and Cd^2+^), underscoring the selective performance of this sensor for Hg(ii) ions. Additionally, the sensor was applied to real water samples to detect mercury ion content and showed significant applicability.^126^ In a comparable approach, E. B. Azimi *et al.* developed boron-doped g-C_3_N_4_ (BCN) as a fluorescent probe for the detection of mercury and ferric ions in real samples. B-doping modulated the electronic structure of g-C_3_N_4_, introducing acid-base functionalities through amine and boron groups that enhance metal ion interactions on its surface. The BCN exhibited strong fluorescence (280/348 nm excitation/emission) that was selectively quenched by Hg^2+^ and Fe^3+^, while inducing a negligible response to other competing ions. In the pH range 7–10, the probe demonstrated a LOD of 185 nM for Hg^2+^ and 154 nM for Fe^3+^, highlighting its reasonable sensitivity for practical analysis. A Stern–Volmer plot led to a plausible quenching mechanism being established, in which quenching by Hg^2+^ ions is attributed to the heavy atom effect and weak covalent interactions with the surface nitrogen donor. In contrast, Fe^3+^ ions probably induced quenching due to their higher affinity and fast chelation with surface N groups. The material's performance was validated to determine Fe^3+^ in spinach and Hg^2+^ in tuna fish, and the results were compared with those obtained by ICP-OES analyses. This strategy is noteworthy because it extends g-C_3_N_4_ applications beyond single-target sensing to dual-ion detection, while maintaining selectivity. The dual response may be advantageous for food and environmental monitoring. However, the detection limits are higher than many reported nanosheet or aptamer-functionalized g-C_3_N_4_ systems, which often achieve sub-nanomolar levels. Moreover, the quenching mechanism relies heavily on non-specific effects, which could reduce selectivity in complex water matrices that are rich in transition metals.^69^

A comparative evaluation of reported g-C_3_N_4_-based mercury sensors (Tables 1–6) reveals that sensor performance is strongly directed by both composite material and sensing mechanism. Fluorescence-based platforms, particularly those relying on quenching or turn-on responses of g-C_3_N_4_ quantum dots and nanosheets, are widely investigated owing to their operational simplicity, rapid response, and high sensitivity. For example, SCNQDs, T-gCNQDs, and g-C_3_N_4_ nanosheets achieve LODs in the range of sub-nanomolar to picomolar. Moreover, underscoring the critical role of heteroatom doping (sulfur-doped C_3_N_4_ tube bundles, S–g-C_3_N_4_) could amplify Hg^2+^ sensor interactions. Further, integration of g-C_3_N_4_ with noble metals, metal oxides, and conductive polymers (g-C_3_N_4_/CeO_2_, g-C_3_N_4_–CdS–CuO, NiFe_2_O_4_/G-C_3_N_4_, V_2_O_5_/gCN, P-doping hollow-tube polymeric C_3_N_4_, and g-C_3_N_4_/chitosan) could exhibit better optical responses than their counterparts in terms of LOD and linear dynamic range. Further strategies, such as photoelectrochemical and electrochemiluminescence, could enhance sensitivity by minimizing background interference and improving signal-to-noise ratios, for instance, g-C_3_N_4_-CdS–CuO and Ru-QD-based hybrid systems.

**Table 1:** An overview of g-C_3_N_4_ nanosheets and g-C_3_N_4_ NP sensors for mercury sensing

S. no.	g-C_3_N_4_ nanosheets & NPs	Sensing approach	Linear range	LOD	Ref.
1	g-C_3_N_4_ nanosheets	Electrochemical	—	9.1 × 10^11^ M	65
2	CNNPs	Fluorescence quenching	0.1–8 and 8–32 µM	0.094 µM	74
3	utg-C_3_N_4_	Electrochemical	0.1–15 µg L^−1^	0.023 µg L^−1^	66
4	g-C_3_N_4_ nanosheets	Fluorescence turns on	—	37 nM	70
5	g-C_3_N_4_	Fluorescence quenching	0.5 to 100 nM	0.17 nM	68
6	GCNNS	Fluorescence quenching	0.001 to 1.0 µM	0.3 nM	73
7	g-C_3_N_4_	Fluorescence quenching	—	6.2 × 10^−7^ M	71
8	T/G-C_3_N_4_	Fluorescence quenching	0–1.25 × 10^3^ nM	27 nM	72
9	CNNPs	Fluorescence quenching	—	60 nM	59

**Table 2:** An overview of g-C_3_N_4_@ metal nanoparticle sensors for mercury detection

S. no.	g-C_3_N_4_@metal NPs	Sensing approach	Linear range	LOD	Ref.
1	g-C_3_N_4_/PtNPs	Colorimetric	—	1.23 nM	78
2	Au@S–g-C_3_N_4_	Fluorometric	100–500 nM	0.275 nM	82
3	g-C_3_N_4_–Au	Fluorescence Turn on	5 to 500 nM	3.0 nM	83
4	Pt/g-C_3_N_4_/PTh NCs	Electrochemical	—	0.009 nM	81
5	PdNPs/g-C_3_N_4_	Electrochemical	0.01–15 µg L^−1^	0.009 µg L^−1^	87
6	Pt/g-C_3_N_4_/PAn NCs	Electrochemical	1–500 nM	0.014 nM	88
7	AuNPs/mpg-C_3_N_4_	Electrochemical	1–25 mg L^−1^	0.103 mg L^−1^	89
8	C_3_N_4_/Ag/ZnWO_4_	Electrochemical	0 nM to 2 mM	0.23 nM	90
9	AgNPs/GO/g-CN	Electrochemical	10 to 180 µL	0.01986 ppm	91
10	Au NPs@g-C_3_N_4_	Photoelectrochemical	1 pM to 1000 nM	0.33 pM	19
11	g-C_3_N_4_ NSs	Fluorescence quenching	0.01–600 nM	5 pM	79

**Table 3:** An overview of g-C_3_N_4_@metal oxide sensors for mercury detection

S. no.	g-C_3_N_4_@metal oxide	Sensing approach	Linear range	LOD	Ref.
1	g-C_3_N_4_/CeO_2_	Colorimetric assay	0.50 nM to 800 nM	0.23 nM	92
2	Mn_3_O_4_/g-C_3_N_4_ composite	Electrochemical	—	0.003 µM	93
3	NiFe_2_O_4_/G-C_3_N_4_	Electrochemical	10 to 800 nM	2.49 nM	94
4	V_2_O_5_/gCN	Electrochemical	0.2 to 100 µM	9.2 nM	95
5	Ti_3_C_2_/Fe_3_O_4_/g-C_3_N_4_	Electrochemical	0.5 to 0.005 µM	0.12 nM	96
6	MnO_2_@g-C_3_N_4_@SPCE	Electrochemical	—	2.6 nM	97
7	Pr/B co-doped g-C_3_N_4_	Electrochemical	0.25–800 nM	42.4 nM	98

**Table 4:** An overview of g-C_3_N_4_ QD sensors for mercury detection

S. no.	g-C_3_N_4_ QDs	Sensing approach	Linear range	LOD	Ref.
1	g-CNQDs	Fluorescence quenching	—	∼10^−9^ M	100
2	g-CNQDs	Chemiluminescence quenching	0.25–10 ng mL^−1^	0.08 ng mL^−1^	4
3	OS-GCNQDs	Fluorescence quenching	0.001–20.0 mM	0.37 nM	101
4	CNQDs	Fluorescence quenching	0.1–10 and 10–30 µM	0.14 µM	102
5	g-C_3_N_4_/CQDs	Photoluminescence	—	8 ng L^−1^	103
6	CdSeQDs/g-C_3_N_4_	Fluorescence quenching	0–32 nmol L^−1^	5.3 nmol L^−1^	104
7	SCNQDs	Fluorescence quenching	10 nM to 1 µM	0.01 nM	105
8	g-CNQD	Fluorescence quenching	0.20 to 21 µM	3.3 nM	106
9	T-gCNQDs	Fluorescence quenching	1.0 to 500 nM	0.15 nM	107
10	DTG-CNQDs	Fluorescence quenching	0.020–0.50 µM	0.63 nM	67
11	g-C_3_N_4_@CdS/FTO	Photoelectrochemical	20–550 nM	12 nM	109
12	ZIF-8@g-CNQD/CdTe	Fluorescence quenching	0.2–3.5 µM	∼46 nM	110
13	Ru-QDs@SiO_2_	Electrochemiluminescence	0.1 nM–10 µM	33 pM	111
14	Ag–S–gCN QDs	Fluorescence quenching	0.1–0.6 µM	0.13 µM	76
15	g-CNQDs	Fluorescence quenching	0–0.1 µmol L^−1^	0.038 µmolL^−1^	112
16	NH_2_-UiO-66/g-CNQDs	Fluorescence quenching	—	2.4 nmol L^−1^	77
17	CNQDs/CNNNs	Fluorescence quenching	0.025–4.0 µmol L^−1^	7.82 nmol L^−1^	114
18	CNNPs-CdTe_0.16_S_0.84_ QDs	Fluorescence quenching	0.05 to 2525 nM	0.009 nM	8

**Table 5:** An overview of g-C_3_N_4_@carbon-based sensors for mercury detection

S. no.	g-C_3_N_4_@carbon-based material	Sensing approach	Linear range	LOD	Ref.
1	P-CN T60/MWCNT/CFE	Electrochemical	2.2 × 10^−11^–8.5 × 10^−6^ mol L^−1^	8.0 × 10^−12^ mol L^−1^	115
2	O-MWCNTs @ p-g-C_3_N_4_	Electrochemical	—	8–60 ng L^−1^	116
3	Sulfur-doped C_3_N_4_ tube bundles	Electrochemical	—	0.61 nM	117
4	P-doping hollow-tube polymeric C_3_N_4_	Fluorescence quenching	—	1.14 nM	118

**Table 6:** An overview of g-C_3_N_4_@polymers and other materials for mercury sensing

S. no.	g-C_3_N_4_@polymer and other materials	Sensing approach	Linear range	LOD	Ref.
1	g-C_3_N_4@_ion imprinted polymer	Electrochemical	0.06 to 25.0 nM	18 pM	120
2	g-C_3_N_4_/chitosan composite	Electrochemical	1.0 × 10^−6^ to 8.0 × 10^−5^ mol L^−1^ and 1.0 × 10^−7^ to 5.0 × 10^−6^ mol L^−1^	1.0 × 10^−8^ mol L^−1^	119
3	P-DPCN	Fluorescence quenching	0–100 µmol L^−1^	0.74 nmol L^−1^	121
4	P-doped carbon nitride oligomer	Fluorescence	1–100 nmol L^−1^	0.35 nmol L^−1^	122
5	g-C_3_N_4_-CdS–CuO	Photoelectrochemical	—	0.84 pM	123
6	O-GCN@PTD-SPCE	Electrochemical	0.05–390 nM	13 pM	25
7	Polymeric g-C_3_N_4_ nanosheets	—	—	0.6 mg L^−1^	10
8	C-g-C_3_N_4_	Electrochemical	5000.0 to 5.0 nM	8.5 pM	124
9	Sulfur-doped g-C_3_N_4_	Electrochemical	0–25 ppb	0.175 ppb	125
10	S-g-C_3_N_4_	Electrochemical	1 × 10^−7^ to 1 × 10^−2^ mol L^−1^	6.5 × 10^−8^ mol L^−1^	126
11	B-g-C_3_N_4_	Fluorescence	—	185 nM	69

## 4. Challenges and future prospects

Despite significant advances in g-C_3_N_4_-based sensors for Hg^2+^ detection, many challenges remain that must be addressed to enable their practical and large-scale deployment. One major constraint associated with current approaches is the trade-off between ultra-low LODs and operating robustness. Although many fluorescence and electrochemical systems exhibit picomolar or sub-nanomolar LODs, these performances are often achieved under controlled laboratory conditions, with limited validation in complex real-water matrices. Furthermore, matrix interference includes coexisting metal ions, such as Fe^3+^, natural organic matter, and other sulfur species, and variable pH remains insufficiently explored across many reported systems, which limits the environmental applicability. Another analytical challenge lies in selectivity and mechanistic transparency. Owing to strong Hg–N/S interactions or heavy-atom effects, Hg^2+^-induced fluorescence quenching occurs, but its definitive mechanistic validation using advanced spectroscopic or theoretical tools is often lacking. Correspondingly, despite superior sensitivity, electrochemical sensors face limitations, for instance, signal drift, electrode fouling, and poor long-term stability, particularly when DNA-based systems, noble metals, or multi-component hybrids are employed.

From a future perspective, rational material design will be central to further performance enhancement. Controlled heteroatom co-doping and molecular-level functionalization of g-C_3_N_4_ could provide well-defined active sites with enhanced Hg^2+^ affinity and improved selectivity. Integrating g-C_3_N_4_ with emerging conductive supports (*e.g.*, MXenes, MOFs, or carbon aerogels) may further improve charge transport and signal amplification in electrochemical and photoelectrochemical platforms. Importantly, future research should prioritize real-world validation, including long-term stability studies, sensor reusability, on-site testing, and compliance with regulatory limits for drinking water. The integration of g-C_3_N_4_-based sensors into portable and wearable analytical devices, coupled with smartphone-based readout or microfluidic platforms, represents a promising direction toward field-deployable mercury monitoring.

## 5. Conclusion

In conclusion, this review article provides the scientific community with an in-depth view of g-C_3_N_4_-based sensing platforms for mercury ions (Hg^2+^). Based on an extensive comparative analysis of recent literature, it is evident that hybrid architectures, including g-C_3_N_4_ NPs, g-C_3_N_4_@metal nanoparticles, g-C_3_N_4_@metal oxides, g-C_3_N_4_@quantum dots, and g-C_3_N_4_@polymeric composites, have emerged as significant, sensitive, and selective sensing materials for mercury. The inherent advantages associated with g-C_3_N_4_, including chemical stability, porous surface area with rich chemistry, facile functionalization, and tunable electronic structure, enable its successful integration, resulting in diverse sensing architectures. Further, structural modulation *via* heteroatom doping and composite formation *via* integration of other materials plays a decisive role in enhancing Hg^2+^ affinity, signal amplification, and selectivity. Nevertheless, despite remarkable laboratory-scale sensitivity and selectivity, some challenges need to be considered, including interference from metal ions, organic materials, and sulfur-related species, in addition to real-sample applicability in complex matrices, long-term stability (*e.g.*, DNA-based systems), and reproducibility.

## Conflicts of interest

There are no conflicts to declare.

## Data Availability

No primary research results, software or code have been included and no new data were generated or analysed as part of this review.
